# Enhancing Post‐Exercise Oxygen Kinetics Modeling With Physiological Bounds and Manual V̇O_2___baseline_ Input: A Novel Approach

**DOI:** 10.1002/ejsc.12306

**Published:** 2025-04-22

**Authors:** Süleyman Ulupınar, İzzet İnce, Cebrail Gençoğlu, Serhat Özbay, Salih Çabuk

**Affiliations:** ^1^ Faculty of Sports Sciences Erzurum Technical University Erzurum Türkiye; ^2^ Faculty of Sports Sciences Ankara Yıldırım Beyazıt University Ankara Türkiye

**Keywords:** alactic contribution, exponential model, oxygen kinetics, post‐exercise

## Abstract

This study addresses a critical limitation in existing computational tools for modeling post‐exercise oxygen consumption kinetics (V̇O_2_). Although exponential modeling provides practical insights into recovery dynamics, the inability to incorporate an individual's pre‐exercise baseline oxygen consumption value (V̇O_2___baseline_) can lead to inaccurate interpretations. A user‐defined baseline allows for more precise modeling by aligning recovery kinetics with the true physiological endpoint, representing the individual's actual recovery target after a sufficient rest. To overcome this limitation, this study employs a customized Python algorithm that incorporates user‐defined baseline V̇O_2_ and uses both mono‐exponential and bi‐exponential models, aiming to improve upon existing analytical methods. Twenty‐two male amateur soccer players participated in this study and performed a 30‐s Wingate test. V̇O_2_ was measured continuously before, during, and after exercise via a metabolic gas analyzer. Both mono‐exponential and bi‐exponential models were used to analyze post‐exercise V̇O_2_ kinetics. The analysis was performed using Origin software (as the reference tool), GedaeLab (a specialized web‐based platform), and a custom‐developed Python algorithm. The bi‐exponential model demonstrated superior fit compared to the mono‐exponential model with higher determination coefficient (*R* ^2^) values. Specifically, *R* ^2^ values were 0.963 ± 0.013 and 0.805 ± 0.078 for the bi‐exponential and mono‐exponential models, respectively. The bi‐exponential model also provided a more accurate approximation of real post‐exercise oxygen consumption integrals at both 5 min and 15 min. Additionally, variations in V̇O_2_baseline_ values had different impacts on key parameters in both models, showing that higher V̇O_2_baseline_ values generally improved the model fit in the mono‐exponential model but had minimal impact on the bi‐exponential model.

**Summary**

- Post‐exercise oxygen kinetics are crucial for evaluating cardiovascular and metabolic efficiency in both athletes and clinical populations.
- Existing computational models often overlook essential physiological parameters, such as baseline oxygen consumption, maximum oxygen consumption, recovery phase duration, and transition time delay, leading to inaccuracies in post‐exercise oxygen kinetics interpretations.
- A Python‐based algorithm has been developed to provide faster and more accurate results by incorporating validated physiological ranges and customizable parameters, ensuring adaptability to diverse populations and research needs.

## 1 Introduction

In the field of exercise physiology, the modeling of intra‐exercise and post‐exercise oxygen kinetics assumes a critical role for a multitude of substantiated reasons (Bertuzzi et al. 2016; Rossiter et al. 1999, 2002). The quantification of V̇O_2_ kinetics serves as an indispensable marker for evaluating the efficacy of cardiovascular and metabolic systems (Artioli et al. 2012; Mullis et al. 1999; Zignoli et al. 2019). Such quantification provides invaluable data regarding the aptitude of the cardiovascular and respiratory apparatus to both deliver and utilize oxygen within active muscular tissue (Rossiter et al. 1999, 2002). This method is especially important for athletes aiming for peak performance and for patients in clinical settings undergoing cardiovascular or respiratory rehabilitation (Chiappa et al. 2008; Monteiro et al. 2020). Additionally, the detailed scrutiny of post‐exercise oxygen kinetics offers the potential to illuminate the fundamental mechanisms that govern exercise‐induced fatigue, energy utilization, and rates of physiological recovery (Mullis et al. 1999; Özyener et al. 2001). Among the most intricate aspects of these analyses is converting the post‐exercise oxygen uptake into a usable model, particularly when considering the contributions of different energy systems. The PCr‐La‐O_2_ method addresses this challenge by quantifying energy contributions from the phosphagen, lactic, and oxygen‐dependent pathways. This methodology leverages post‐exercise oxygen kinetics to estimate alactic energy expenditure, providing a comprehensive understanding of energy system interactions during and after exercise (Artioli et al. 2012; Bertuzzi et al. 2016; Kaufmann et al. 2022). Consequently, advanced computational algorithms proficient in precisely modeling these oxygen kinetics avail medical practitioners, exercise physiologists, and athletes with a nuanced instrument for the individualization of exercise protocols, the assessment of health metrics, and the fine‐tuning of therapeutic modalities (Beneke et al. 2002; Zignoli et al. 2019).

Exponential models play a critical role in a wide range of scientific disciplines, extending far beyond exercise physiology. They are essential for understanding radioactive decay in physics, population growth in biology, interest rates in economics, and drug kinetics in medicine (Chevallier et al. 2019; Cordovil et al. 2005; Xu et al. 2019). These models also find applications in diverse fields such as ecology, neuroscience, and epidemiology (Adair et al. 2010; Easton 2005). Given the diverse applications of exponential models across various scientific disciplines, it is somewhat surprising that specialized methodologies for modeling V̇O_2_ kinetics in existing softwares are conspicuously lacking (Artioli et al. 2012; Bertuzzi et al. 2016). Despite a robust body of literature providing extensive data on key physiological parameters—such as resting oxygen consumption, maximum oxygen uptake, and ATP‐PCr replenishment rates—the fitting of these models to empirical data often occurs in a vacuum of such information (Chaabène et al. 2012; Özbay and Ulupınar 2022). Therefore, it stands to reason that a novel approach, which incorporates defining the ranges for parameters within the model based on our current body of knowledge, would yield more useful and accurate outcomes. This integrated approach would not only align the model with existing empirical data but also potentially offer greater predictive accuracy and clinical applicability.

In the immediate post‐exertional phase subsequent to high‐intensity physical activity, a marked augmentation in pulmonary oxygen uptake (V̇O_2_) is observed (Ozkaya et al. 2024; Vickery‐Howe et al. 2024). Initiating from a quiescent baseline typically situated between 200 and 500 mL/min, V̇O_2_ escalates to the maximal attainable level for the individual, occasionally exceeding 5000–6000 mL/min under extreme conditions. Following exercise cessation, off‐kinetics refers to the process by which elevated oxygen uptake and ventilation gradually return to baseline levels (Bertuzzi et al. 2016; Rossiter et al. 1999, 2002). This recovery, often described as the “rapid phase,” occurs within a time frame of 30 s to a few minutes and is influenced by factors such as exercise intensity and individual fitness levels. Athletes with higher fitness levels are capable of repaying larger oxygen deficits within the same time frame, demonstrating more efficient recovery dynamics (Laforgia et al. 2006; Panissa et al. 2021; Rosvoglou et al. 2023). Conversely, the “slow” phase unfolds over a more protracted time frame, potentially extending to several hours, and is influenced by the preceding exercise's intensity and duration (Kaufmann et al. 2022; Panissa et al. 2021; Rossiter et al. 2002). This phase is characterized by a multifaceted physiological response aimed at restoring baseline conditions. This involves lactate clearance, normalization of mitochondrial metabolic rates, and the gradual return of elevated heart rate, respiratory frequency, and core body temperature to their pre‐exercise states, alongside the modulation of catecholamine levels such as epinephrine and norepinephrine (Jentjens and Jeukendrup 2003; Reilly and Ekblom 2005). Moreover, this slow phase is implicated in the metabolic conversion of lactate to glucose, a process termed gluconeogenesis (Jentjens and Jeukendrup 2003).

Despite the well‐documented nature of these kinetic phases in empirical studies, current computational methodologies designed to clarify post‐exercise oxygen kinetics often neglect some essential components (Artioli et al. 2012; Kaufmann et al. 2022; Ulupınar et al. 2021). Typically, these models are calibrated solely to align with empirical data while disregarding established physiological parameters such as baseline and maximal V̇O_2_ (Kaufmann et al. 2021; Özyener et al. 2001; Rossiter et al. 1999). In practice, baseline V̇O_2_ values are routinely measured prior to exercise and used to estimate aerobic contributions during exercise. However, existing programs do not allow these baseline measurements to be integrated into exponential modeling. Such limitations in existing computational models can lead to physiologically inconsistent and potentially misleading outcomes. For example, baseline V̇O_2_ values that should be around 300 mL/min may be miscalculated as 900 mL/min, distorting critical parameters like amplitude and tau (Hill 2023; Özyener et al. 2001). Additionally, some models generate implausible results, such as negative baseline V̇O_2_ values or significantly low *R* ^2^ values, which undermine the validity of the model fits (Figures S5–S7). These issues highlight the critical need for a more robust and physiologically grounded approach to accurately model post‐exercise oxygen kinetics. To address these critical limitations, this study introduces a Python‐based algorithm that integrates user‐defined baseline VO_2_ values within both mono‐exponential and bi‐exponential models. Unlike existing computational approaches, this novel algorithm incorporates validated physiological parameter ranges, ensuring that modeled outputs align with established physiological realities. By enabling precise control over baseline VO_2_ inputs and applying robust modeling techniques, this study aims to provide a more accurate and reliable tool for analyzing post‐exercise oxygen kinetics. Such an approach holds potential benefits for both athletic performance assessment and clinical applications, offering deeper insights into recovery dynamics and energy system contributions.

## 2 Methods

### 2.1 Experimental Design

The study utilized a two‐visit experimental design for the comprehensive evaluation of post‐exercise oxygen kinetics. All assessments were conducted in an environmentally controlled setting, with temperature and humidity maintained at 20°C–23°C and 50%–55%, respectively. During the familiarization visit, participants were acclimated to the experimental procedures and apparatus, particularly the respiratory mask and maximal cycle ergometer (Peak Bike 894e, Monark, Sweden).

On the experimental visit, baseline oxygen consumption (V̇O_2___baseline_) was measured while participants maintained a seated posture for a 10‐min period to ensure complete relaxation and minimize muscle activity. The average V̇O_2_ from the final five‐minute segment was used to set the baseline. Following this, participants performed a standardized warm‐up on the ergometer. The warm‐up consisted of five bouts of 30 s each at 100 W, alternating between 20 s at 60 rpm and 10 s at 110 rpm. This protocol was designed to physiologically prepare participants for the Wingate test and to standardize the conditions across trials.

Participants then performed a 30‐s Wingate test using a load equivalent to 10% of their body mass. The Wingate test is widely regarded as a reference exercise protocol for evaluating energy system contributions due to its ability to simultaneously engage all three energy systems (Beneke et al. 2002; Bogdanis et al. 1996; Julio et al. 2019; Tortu et al. 2024). Research has consistently demonstrated that this short‐duration, high‐intensity exercise elicits the significant activation of anaerobic metabolism while also involving aerobic pathways during exercise (Julio et al. 2019; Smith and Hill 1991). As such, the Wingate test provides an ideal opportunity to examine post‐exercise oxygen kinetics (off‐kinetics), particularly in the context of anaerobic load and recovery dynamics.

Real‐time oxygen consumption was captured before, during, and after the exercise using a portable metabolic gas analyzer (COSMED K5, Rome, Italy). The gas analyzer was calibrated prior to each session using known gas concentrations and flow rates to ensure accuracy. Immediately after completing the test, participants ceased pedaling and returned to a seated posture on a chair. V̇O_2_ kinetics were monitored continuously for an additional 15 min during this recovery phase while participants remained seated.

To calculate total oxygen consumption during the 15‐min recovery period, the breath‐by‐breath data collected by the gas analyzer were integrated over time using the trapezoidal method. This approach allowed for precise calculation of the total oxygen consumed during recovery. The integral values for the initial 5‐min segment and the full 15‐min period were computed separately to assess the dynamics of fast and slow recovery phases. This standardized recovery protocol was implemented to minimize external influences and ensure consistency across participants.

For data analysis, both mono‐exponential and bi‐exponential models were employed. The analyses were performed using Origin software (OriginLab Corporation, Northampton, MA, USA) as the reference tool, complemented by GedaeLab (Bertuzzi et al. 2016)—a specialized web‐based application for V̇O_2_ kinetics—and a purpose‐developed Python‐based algorithm.

### 2.2 Participants

The study was conducted with 22 male amateur soccer players (age: 21.68 ± 2.12 years; height: 173.95 ± 4.55 cm; weight: 69.09 ± 3.13 kg) who voluntarily participated. These participants were recruited from various local soccer clubs and met specific inclusion and exclusion criteria. To be eligible for inclusion in the study, participants had to be males aged between 18 and 25 years, have at least 5 years of soccer training experience, and be currently engaged in soccer training at least three times per week. Additionally, they must have been free of any known cardiovascular, respiratory, or metabolic diseases. Exclusion criteria included a history of major orthopedic injuries within the past 6 months, current use of medication that could affect cardiovascular or metabolic responses, or an inability to complete all aspects of the study's testing protocol.

### 2.3 Development of Python‐Based Algorithm

#### 2.3.1 Data Collection and Preprocessing

Data were collected using a portable gas analyzer. All collected data were saved in Excel files. The focus of our analysis was primarily on time and oxygen consumption (V̇O_2_).

#### 2.3.2 Software and Libraries

Analysis was performed using Python (V.3.7.0) with libraries such as Pandas for data manipulation, SciPy for optimization and curve fitting, NumPy for numerical operations, and Matplotlib for data visualization (Appendix 4, Python codes).

#### 2.3.3 Data Validation and Cleaning

The program asks the user to input the directory path and file name. It validates the file format and prompts the user to specify the column names for time and V̇O_2_ data. Time data are then converted to seconds, and duplicate time entries are filtered.

#### 2.3.4 Data Smoothing

An optional Savitzky–Golay filter (with a 5‐point window) was applied to reduce noise in the V̇O_2_ data. This technique was selected for its ability to smooth the data while preserving the original shape and important features of the signal, such as peaks and slopes. Unlike traditional moving average methods, the Savitzky–Golay filter uses polynomial fitting within a sliding window, which helps maintain the physiological integrity of the data while minimizing random fluctuations.

#### 2.3.5 Data Interpolation

For datasets with missing time points or discontinuities, the algorithm offers an optional cubic spline interpolation method. This technique was selected due to its ability to generate a smooth curve that passes through all known data points, thus maintaining the physiological trends of V̇O_2_ kinetics during recovery. Cubic spline interpolation provides a balance between maintaining the fidelity of the raw data and filling in gaps without introducing significant bias.

#### 2.3.6 Curve Fitting

Two types of curve fitting methods are implemented:

*Mono‐exponential Model*: Describes V̇O_2_ response with parameters V̇O_2_baseline_, A1, td, and τ1.

*Bi‐exponential Model*: Adds a slow component to describe V̇O_2_ kinetics using parameters V̇O_2_baseline_, A1, td, τ1, A2, and τ2.

(1) $$\dot{V}O_{2\;\left(t\right)}=\dot{V}O_{2\_\text{baseline}}+A\;\left[e^{–\;\left(t\;–\;\text{td}\right)\;/\;\tau }\right]$$

(2) $$\dot{V}O_{2\;\left(t\right)}=\dot{V}O_{2\_\text{baseline}}+A_{1}\;\left[e^{–\;\left(t\;–\;\text{td}\right)\;/\;\tau 1}\right]+A_{2}\;\left[e^{–\;\left(t\;–\;\text{td}\right)\;/\;\tau 2}\right]$$

V̇O_2_baseline_ is the level of oxygen consumption at rest. A_1_ parameter represents the amplitude of the fast phase and higher A_1_ value indicates that the fast phase is more prominent. A_2_ parameter represents the amplitude of the slow phase and higher A_2_ value indicates that the slow phase is more prominent. “td” is known as “time delay,” and this parameter represents the time elapsed after the cessation of exercise and before the commencement of the fast and slow phases of V̇O_2_ kinetics during recovery. τ_1_ (tau_1_) is the time constant for the fast phase, indicating how quickly the fast phase occurs and lower τ_1_ value suggests a faster fast phase. τ_2_ (tau_2_) is the time constant for the slow phase, indicating how quickly the slow phase occurs and lower tau_2_ value suggests a faster slow phase.

Curve fitting is subject to adaptive bounds and initial conditions, allowing for the manual entry of known V̇O_2_baseline_ values for more accurate amplitude and tau parameters.

#### 2.3.7 Model Evaluation

Evaluation includes *R* ^2^, Akaike information criterion (AIC), and bias‐corrected Akaike information criterion (AICc) along with additional *R* ^2^ values focused on the fast phase, which is the focal point of the PCr‐La‐O_2_ method (the first 5 min post‐exercise were used as a sample).

#### 2.3.8 Energy Unit Conversion

Calculates energy production in both kcal and kJ.

#### 2.3.9 Advantageous Features

*User Flexibility:* Allows for specific column names and optional data smoothing and interpolation.

*Adaptive Bounds:* Curve fitting adapts based on user‐defined V̇O_2_baseline_ and parameter bounds.

*Comprehensive Model Evaluation:* Incorporates *R* ^2^, AIC, and AICc metrics. Additionally, offers *R* ^2^ values specifically for the fast phase of exercise (the first 5 min post‐exercise were used as a sample).

*Simultaneous Curve Visualization:* Presents both mono‐exponential and bi‐exponential curves simultaneously and distinguishes between fast and slow phases in the bi‐exponential model.

*Support for Absolute and Relative V̇O*_*2*_ *Values:* Capable of detecting both absolute and relative V̇O_2_ values and adjusts calculations and graphical representations accordingly.

*Estimated Energy Contribution of Alactic Pathway:* Offers an estimated energy contribution, a feature not found in Origin.

*Integral Values:* Provides integral values both for the actual data and the model curves.

*Time Efficiency:* Achieves 10–20 times faster results when smoothing and interpolation processes are applied, offering significant time savings.

The algorithm runs on various development environments, including Jupyter Notebook, PyCharm, Visual Studio Code, and Google Colab, providing flexibility to the researchers according to their preferred setup. The project code is available on GitHub (https://github.com/XX/FitExpVO2_Modeling‐of‐Post‐exercise‐Oxygen‐Kinetic).

### 2.4 Statistical Analyses

Statistical analysis was conducted using a multitool approach to ensure a comprehensive understanding of V̇O_2_ kinetics. Origin software and GedaeLab, a specialized online platform for modeling VO_2_ kinetics, were utilized to create example graphs and demonstrate how V̇O_2_baseline_ values and *R* ^2^ estimates generated by these tools can deviate from physiological reality. These tools served as references to highlight the limitations of existing models in comparison to the custom‐built Python algorithm. The Python algorithm incorporated user‐defined V̇O_2_baseline_ values and validated physiological parameter bounds, providing a more accurate and robust modeling framework. To compare parameters between the mono‐exponential and bi‐exponential models, paired sample *t*‐tests were employed. The practical significance of these comparisons was assessed using Cohen's d. Repeated measures one‐way ANOVA was used for additional comparative analysis of integral values obtained from actual data and model predictions. All data are presented as the mean and standard deviation, with statistical significance set at a *p*‐value threshold of 0.05. Data visualization was carried out using GraphPad software, completing the multitool approach to statistical analysis.

## 3 Results

### 3.1 Model Fitting Analyses

Fitting analyses were conducted to elucidate the specific parameters for both the mono‐exponential and bi‐exponential models. Both models utilized a common V̇O_2_baseline_, measured at 384.4 ± 56.2 mL. For the mono‐exponential model, the amplitude was 2760.6 ± 388.0 mL, the time delay was 3.69 ± 1.16 s, and the tau was 162.3 ± 36.6 s. The bi‐exponential model yielded a fast‐phase amplitude of 2505.5 ± 466.9 mL (*p* < 0.001), a time delay of 2.70 ± 1.90 s (*p* < 0.001), and a fast‐phase tau of 65.3 ± 12.8 s (*p* < 0.001). The bi‐exponential model also revealed a slow‐phase amplitude of 840.7 ± 270.3 mL and a slow‐phase tau of 742.9 ± 187.1 s (*p* < 0.001).

### 3.2 Model Comparisons and Statistical Parameters

The study assessed the fit of mono‐exponential and bi‐exponential models to the post‐exercise oxygen consumption data using a comprehensive set of statistical parameters. As presented in Table 1, the bi‐exponential model provided a significantly better fit to the data compared to the mono‐exponential model. The determination coefficient (*R* ^2^) was significantly higher in the bi‐exponential model (0.963 ± 0.013) compared to the mono‐exponential model (0.805 ± 0.078, *p* < 0.001). Furthermore, when focusing on the first 5 min post‐exercise (*R* ^2^_5min_), the bi‐exponential model still outperformed the mono‐exponential model with values of 0.957 ± 0.016 and 0.880 ± 0.046, respectively (*p* < 0.001). The AICc also indicated a better fit for the bi‐exponential model (4745 ± 181) compared to the mono‐exponential model (5553 ± 227, *p* < 0.001). A higher *R* ^2^ and a lower AICc value indicate a better fit of the model to the data. In terms of alactic energy contribution measured in kJ, the bi‐exponential model estimated a significantly lower value (57.8 ± 16.5 kJ) compared to the mono‐exponential model (160.4 ± 32.1 kJ, *p* < 0.001). These results collectively demonstrate the superior explanatory power of the bi‐exponential model over the mono‐exponential model in describing post‐exercise V̇O_2_ kinetics.

**TABLE 1:** Fit statistics for the mono‐ and bi‐exponential model.

	Mono‐exponential	Bi‐exponential	*t*	*p*	Cohen's d
*R* ^2^	0.805 ± 0.078	0.963 ± 0.013	10.007	< 0.001	2.83
*R* ^2^_5min_	0.880 ± 0.046	0.957 ± 0.016	8.441	< 0.001	2.24
AICc	5553 ± 227	4745 ± 181	17.845	< 0.001	3.94
Alactic energy contribution (kJ)	160.4 ± 32.1	57.8 ± 16.5	18.282	< 0.001	4.01

*Note: R* ^2^ determination coefficient; *R* ^2^_5min_ determination coefficient for the first 5 min post‐exercise; AICc bias‐corrected Akaike information criterion.

### 3.3 Evaluating the Accuracy of Mono‐ and Bi‐Exponential Models in Post‐Exercise Oxygen Consumption

This study assessed the accuracy of mono‐exponential and bi‐exponential models in approximating the integral values of oxygen consumption for two distinct post‐exercise periods: the initial 5‐min segment and the entire 15‐min recovery period (Table 2). For the initial 5‐min recovery segment, the observed oxygen consumption data revealed an average value of 8.06 ± 0.23 L O_2_ (*p* < 0.001). The mono‐exponential model slightly overestimated this value, providing an average of 8.43 ± 0.24 L O_2_, which was significantly different from the observed (real) data (*p* < 0.001). In contrast, the bi‐exponential model provided an average of 8.10 ± 0.24 L O_2_, closely approximating the observed data with no statistically significant difference (*p* = 0.77). The bi‐exponential model differed significantly from the mono‐exponential model (*p* < 0.001), highlighting its superior accuracy in representing oxygen consumption dynamics during the initial 5‐min recovery period.

**TABLE 2:** Integral statistics for the mono‐ and bi‐exponential model.

	Real data	Mono‐exponential	Bi‐exponential	*F*	*p*	η_p_^2^
Integral_15min_ (L O_2_)	15.57 ± 2.53	13.54 ± 1.91^a^	15.55 ± 2.52^b^	171.5	< 0.001	0.891
Integral_5min_ (L O_2_)	8.06 ± 0.23	8.43 ± 0.24^a^	8.10 ± 0.24^b^	113.2	< 0.001	0.844

^a^ Significantly different from the real data.

^b^ Significantly different from the mono‐exponential.

For the 15‐min recovery period, the observed oxygen consumption data had an average value of 15.57 ± 2.53 L O_2_. The mono‐exponential model significantly underestimated this value, providing an average estimate of 13.54 ± 1.91 L O_2_ (*p* < 0.001). In contrast, the bi‐exponential model closely approximated the observed data, with no statistically significant difference compared to the observed values (average: 15.55 ± 2.52 L O_2_). As expected, the difference between the mono‐exponential and bi‐exponential models was statistically significant. These findings clearly indicate that the bi‐exponential model demonstrates superior accuracy in approximating observed oxygen consumption data for both the initial 5‐min segment and the full 15‐min recovery period post‐exercise.

### 3.4 Impact of V̇O_2_baseline_ Variations on Model Parameters

**FIGURE 1:**
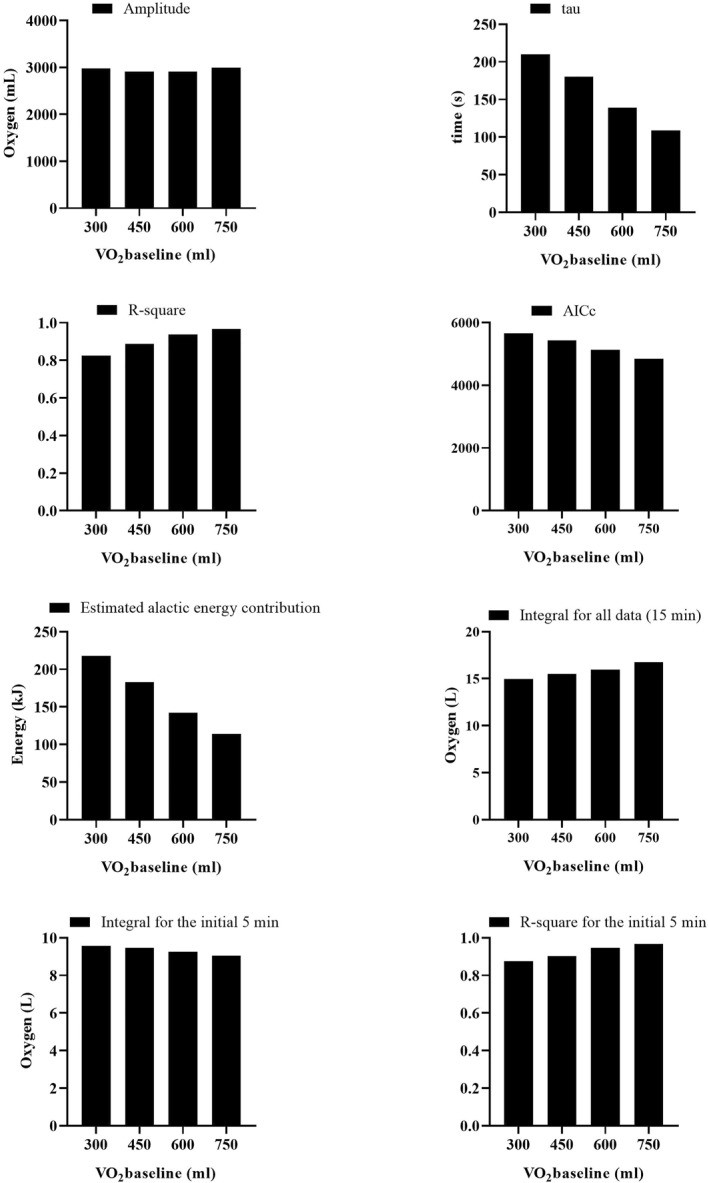
Effects of varied V̇O_2_baseline_ on parameters, model fit, and integral values in mono‐exponential modeling. The presented data are based on the results from a participant serving as a representative example for illustration purposes (see Appendix 1, time1 and oxy1 data for participant 1).

In the mono‐exponential model, variations in the V̇O_2_baseline_ value, ranging from 300 to 750, influenced multiple key parameters (Figure 1). A declining trend was observed in the tau, AICc, and estimated alactic energy contribution as the V̇O_2_baseline_ value increased. On the contrary, the *R* ^2^ coefficient showed an inclining trend, indicating an enhanced model fit with higher V̇O_2_baseline_ values. The amplitude parameter remained largely stable across the spectrum of V̇O_2_baseline_ variations. Furthermore, the 15‐min integral values exhibited a modest increase, whereas the 5‐min integral values demonstrated a slight decreasing tendency as the V̇O_2_baseline_ value escalated.

**FIGURE 2:**
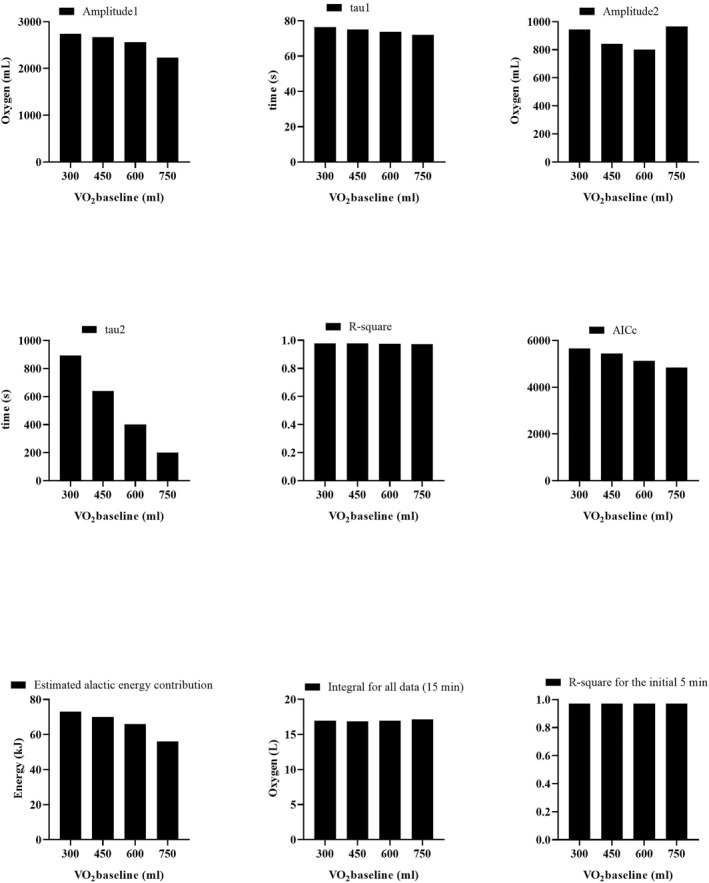
Effects of varied V̇O_2_baseline_ on parameters, model fit, and integral values in bi‐exponential modeling. The presented data are based on the results from a participant serving as a representative example for illustration purposes (see Appendix 1, time1 and oxy1 data for participant 1).

In the bi‐exponential model, a series of key parameters was affected by the manipulation of the V̇O_2_baseline_ value across a range of 300–750 (Figure 2). A declining trend was noticed in amplitude_1_, tau_1_, tau_2_, AICc, and the estimated alactic energy contribution as V̇O_2_baseline_ increased. The *R* ^2^ coefficient and integral values remained relatively stable, resisting significant changes even as V̇O_2_baseline_ values shifted. In contrast, amplitude_2_ did not exhibit a consistent trend and appeared to be conditioned by variations in the other parameters. These observations were based on a representative participant and were largely consistent across the study population (see Appendix 1, Time and oxygen data).

## 4 Discussion

### 4.1 Analysis of Post‐Exercise Oxygen Consumption and Novel Approaches

In the field of exercise physiology, the analysis of post‐exercise oxygen consumption patterns has long been a focal point for enhancing both athletic performance and recovery strategies (Beneke et al. 2002; Özyener et al. 2001; Rossiter et al. 1999; Ulupınar et al. 2021). However, established programs and algorithms (OriginPro and GedaeLab) often lack the flexibility to incorporate user‐defined baseline V̇O_2_ values and rarely offer a comprehensive evaluation that includes metrics like *R* ^2^, AICc, and estimated alactic energy contributions (Artioli et al. 2012; Bertuzzi et al. 2016; Panissa et al. 2018). This study aimed to offer a comprehensive understanding of post‐exercise oxygen kinetics by employing both mono‐exponential and bi‐exponential models for V̇O_2_ kinetics. Utilizing a custom‐developed Python‐based algorithm, we introduced an innovative approach for model fitting that accommodates user‐defined V̇O_2___baseline_ values. This tailored approach not only captures multiple metrics such as *R* ^2^, AICc, and estimated alactic energy contributions but also achieves significant time efficiency through optimized smoothing and interpolation processes.

Although smoothing and interpolation are widely used in analyses involving time‐dependent continuous data, there is no universally accepted standard or consensus on how these processes should be implemented in modeling VO_2_ off‐kinetics. Existing tools such as GEDAELAB lack these options entirely, while Origin requires additional steps to apply such processes effectively. In contrast, our algorithm offers these features as optional tools, empowering users to apply them as needed based on their specific dataset and research goals. This flexibility not only enhances the algorithm's utility but also allows researchers to adapt their approach to the unique demands of their study. To the best of our knowledge, this is the first study to combine these various elements—user‐defined baseline, comprehensive model metrics, and optional smoothing and interpolation—into a single framework, thereby offering a more nuanced and efficient method for V̇O_2_ kinetics analysis.

### 4.2 Comparative Performance of Models

**FIGURE 3:**
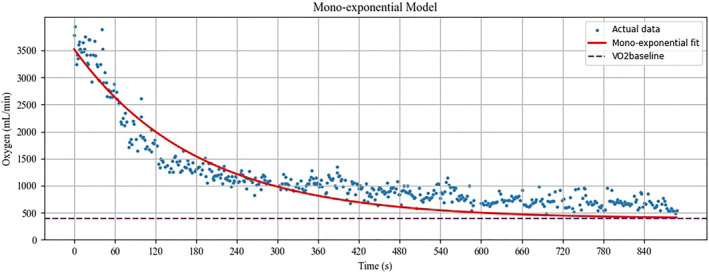
Application of the custom python‐based algorithm for fitting the mono‐exponential model to actual data. The presented data are based on the results from a participant serving as a representative example for illustration purposes (see Appendix 1, time1 and oxy1 data for participant 1).

**FIGURE 4:**
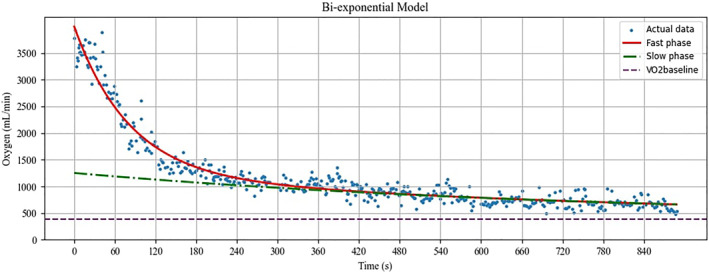
Application of the custom python‐based algorithm for fitting the bi‐exponential model to actual data. The presented data are based on the results from a participant serving as a representative example for illustration purposes (see Appendix 1, time1 and oxy1 data for participant 1).

In our analysis, the bi‐exponential model consistently outperformed the mono‐exponential model in terms of the *R* ^2^ for both the initial 5‐min and the full 15‐min post‐exercise periods (see Figures 3 and 4). However, the mono‐exponential model demonstrated a higher *R* ^2^ value for the initial 5‐min window compared to the full 15‐min period. This discrepancy is likely attributable to the overestimation of the slow phase in the mono‐exponential model, which misaligns with the actual data. In the estimation of energy pathway contributions via the PCr‐La‐O_2_ method, oxygen consumption is measured for a sustained 15‐min period, during which it seldom returns to the baseline. However, it is well‐known that with adequate rest, V̇O_2_ will eventually revert to its baseline level. In scenarios where the mono‐exponential model auto‐calibrates the V̇O_2_baseline_, it often produces an inflated baseline estimate, compromising the validity of the results. Therefore, the ability of our algorithm to incorporate user‐defined V̇O_2_ baseline values presents a significant methodological advantage.

Post‐exercise oxygen consumption is known to eventually return to baseline levels, despite the temporary increase following the cessation of physical activity (Mullis et al. 1999; Özyener et al. 2001; Panissa et al. 2018). Current calculations based on the PCr‐La‐O_2_ method are limited by their reliance on exponential models, which often necessitate the model's own auto‐calibration of the V̇O_2___baseline_. This can lead to inflated or deflated V̇O_2___baseline_ estimates, thereby undermining the estimations' validity. Our custom‐developed Python algorithm was specifically designed to address these limitations by allowing user‐defined V̇O_2___baseline_ values and implementing physiologically informed modeling. When we manually entered an actual V̇O_2___baseline_ value of 390 mL/min into the algorithm, we achieved *R* ^2^ values of 0.805 and 0.963 for the mono‐exponential and bi‐exponential models, respectively (see Figures 3 and 4). This demonstrates a notable improvement in model accuracy, particularly for the bi‐exponential model, which closely approximates the observed data.

In contrast, analyses conducted with reference software generated *R* ^2^ values of 0.949 and 0.957 but calculated the V̇O_2___baseline_ as 780 mL/min for the mono‐exponential model and as low as −1.6 (or zero) for the bi‐exponential model. Similarly, an alternative web‐based software yielded an *R* ^2^ of just 0.490 for the bi‐exponential model and estimated the V̇O_2___baseline_ at a significantly inflated 780 mL/min (see Appendix 3, Model‐fit graphs). Such discrepancies illustrate the limitations of current modeling tools, particularly their reliance on asymptotic assumptions and auto‐calibration. Additionally, Table 1 highlights the superiority of the bi‐exponential model in key fit metrics. For instance, the bi‐exponential model demonstrated a significantly higher *R* ^2^ value compared to the mono‐exponential model, indicating a superior fit to the data. Similarly, the bi‐exponential model's AICc was considerably lower than that of the mono‐exponential model, further confirming its enhanced accuracy in representing the observed data. The bi‐exponential model accurately estimated alactic energy contributions, demonstrating superior accuracy over the mono‐exponential model, which significantly overestimated this metric (*p* < 0.001). These findings highlight the robustness of our custom algorithm in analyzing post‐exercise oxygen kinetics and alactic energy contributions.

### 4.3 Impact of Manual V̇O_2_baseline_ Input on Model Parameters

The accuracy gained by manually inputting V̇O_2_baseline_ values extends its impact to the amplitude and tau parameters as well, as evidenced in our analyses. For instance, Figure 1 illustrates that as the V̇O_2_baseline_ value deviates from its true value by being artificially increased, there is no consistent trend in amplitude, yet a notable decrease in tau values can be observed. Similarly, in Figure 2, artificially inflating the V̇O_2_baseline_ leads to a substantial decline in amplitude_1_ and a slight reduction in tau_1_. To put it in simpler terms, if a true V̇O_2_baseline_ value of 300 mL/min is inaccurately estimated as 900 mL/min, it could reduce the amplitude from 3000 mL/min to 2400 mL/min. Given that tau is a temporal parameter representing 63.2% of the amplitude, it would inevitably also decrease. Such inaccuracies can be consequential, especially in the context of the PCr‐La‐O_2_ method, where amplitude and tau are used to determine contributions from the alactic pathway. Hence, inflated baseline values could lead to underestimations in the alactic energy contribution, adding another layer of error to the study findings.

### 4.4 Superiority of the Bi‐Exponential Model and Estimation of Alactic System Contributions

In this study, the superiority of the bi‐exponential model in capturing post‐exercise V̇O_2_ kinetics was corroborated, particularly in its estimation of alactic system contributions. Although the PCr‐La‐O_2_ method offers practical advantages for estimating energy system contributions or making inferences about the contribution of the phosphagen system based on off‐kinetics, its ability to fully represent physiological processes remains a subject of ongoing debate. However, it is important to note that even the “gold standard” method, muscle biopsy, has its limitations. For instance, muscle biopsy samples typically represent only a localized region of a specific muscle, which may not fully reflect systemic energy dynamics (Bogdanis et al. 1996; Meola et al. 2012). During activities like running or cycling, it is well‐documented that respiratory muscles and upper‐body muscles also contribute to the overall energy expenditure, yet these contributions are often overlooked in localized biopsy analyses. Additionally, the time delay of 10–15 s required to obtain a muscle sample post‐exercise may result in missing the most critical moments of phosphocreatine (PCr) depletion and resynthesis. Elite athletes, in particular, are likely to initiate significant PCr resynthesis during this brief interval, further complicating the accuracy of biopsy‐derived estimates. When the ethical considerations, invasiveness, and logistical challenges of muscle biopsy are taken into account, the practical benefits of methods like the PCr‐La‐O_2_ approach become evident.

In the present study, the bi‐exponential model yielded results (57.8 ± 16.5 kJ) that closely align with previously reported estimates of alactic metabolism contributions during maximal anaerobic tests, which range from 40 to 60 kJ (Kaufmann et al. 2021; Ulupınar and Özbay 2022). These approximations are commonly derived using amplitude and tau values. By contrast, the mono‐exponential model significantly overestimated alactic energy contribution (160.4 ± 32.1 kJ), likely due to the model's overestimation of amplitude resulting from an underestimated V̇O_2___baseline_ value, which artificially elongated the tau value. Although increasing the estimated V̇O_2___baseline_ might improve the mono‐exponential model's fit for 15‐min post‐exercise data, such an adjustment would deviate from the true physiological response and is therefore not justifiable.

### 4.5 Customization of Models for Post‐Exercise Oxygen Kinetics and Parameter Boundaries

Although exponential models are versatile and widely applicable, their customization to meet the specific demands of post‐exercise oxygen kinetics offers a nuanced and strategic avenue for exploration(Adair et al. 2010; Cordovil et al. 2005; Easton 2005). Previous research provides valuable insights into the plausible boundaries of key parameters (Beneke et al. 2002; Özyener et al. 2001; Rossiter et al. 1999, 2002). For instance, previous studies have indicated that in the context of high‐intensity and strenuous exercises, the tau_1_ value could extend up to about 1 minute, whereas tau_2_ values might reach as high as 1000 s(Chorley et al. 2022; Özyener et al. 2001). Amplitude values have generally fluctuated within the range of 1500–3500 mL/min (Artioli et al. 2012; Özyener et al. 2001; Ulupınar and Özbay 2022). Taking into account that post‐exercise recovery often commences from the peak oxygen consumption achieved during the activity, we have set liberal amplitude boundaries in this study, with an upper limit of 100 kg/mL/min, to be inclusive of a broad population (Gao et al. 2021; Polat et al. 2018). It is worth noting that while these bounds have been liberally defined to serve our overarching aim of generalizability, they can be adjusted to more conservative ranges based on the unique characteristics of specific research cohorts. By incorporating these predefined limits into our algorithmic model fitting, we not only minimize the risk of spurious estimations but also bolster the predictive validity of our models.

The concept of time delay in post‐exercise oxygen kinetics is a nuanced subject, particularly in the immediate physiological aftermath of exercise cessation (Poole and Jones 2012; Stirling and Zakynthinaki 2009; Whipp 2009). The rapid phase, characterized by phosphocreatine (PCr) resynthesis and the replenishment of muscle and blood myoglobin stores, is generally considered the dominant factor in elevated oxygen consumption post‐exercise (Burnley and Jones 2007; Özyener et al. 2001; Rossiter et al. 1999). However, contributions from the slower phase, including lactate clearance, catecholamine modulation, and body temperature regulation, should not be overlooked (Jentjens and Jeukendrup 2003; Laforgia et al. 2006). In practical terms, acknowledging the slow phase is crucial for optimizing recovery strategies in both athletic and clinical settings. For instance, training programs can incorporate active recovery protocols to enhance lactate clearance, whereas monitoring catecholamine responses may guide stress management techniques. Furthermore, maintaining an optimal thermal environment post‐exercise could support more efficient body temperature regulation, contributing to overall recovery efficiency. Traditional exponential models, when fitted using existing software tools, often assign a separate time delay parameter specifically for the slow phase (Artioli et al. 2012; Bertuzzi et al. 2016). This can lead to unnecessary complexity and diminish the model's physiological interpretability. An alternative approach favored by some researchers is to omit the initial seconds post‐exercise that indicate time delay and proceed with calculations in the absence of a time delay term (Özyener et al. 2001). The algorithm we have developed in this study aims to streamline this complexity by providing a computational method that employs a single time delay term, effectively capturing the kinetics of both the rapid and slow phases.

### 4.6 Practical Considerations

This study offers valuable contributions to exercise physiology, particularly by enhancing the methodological approaches used in analyzing post‐exercise oxygen consumption through bi‐exponential models. The introduction of a custom Python‐based algorithm, which incorporates user‐defined V̇O_2_baseline_ values, presents an innovative approach with practical implications for both training programs and athletic performance. Policy makers, sports scientists, and practitioners can leverage these insights to optimize athletes' training and recovery strategies and improve performance.

However, several limitations should be acknowledged. The study was conducted exclusively on male soccer players, which may limit its generalizability to other populations, including female athletes, individuals from different sports disciplines, and those with specific medical conditions. Future studies should explore these models across diverse groups to enhance their applicability. Additionally, although the model's parameter bounds (e.g., tau_1_, tau_2_, and amplitude) were set broadly to accommodate a general population, more specific bounds might be needed for elite athletes or specific clinical populations. Although the algorithm is robust, its accuracy depends on the quality of input data, highlighting the need for well‐structured and noise‐free datasets. Furthermore, although the model is grounded in computational principles, empirical validation through controlled physiological experiments would strengthen its reliability.

In summary, although this study provides a solid foundation for advancing personalized sports science, researchers and practitioners should apply these findings with a clear understanding of the study's context and limitations. Future research should aim to validate and refine these models, extending their utility across various sports disciplines, demographic groups, and health conditions. By bridging methodological advancements with practical applications, this study paves the way for more individualized and data‐driven approaches in sports performance and exercise physiology.

## 5 Conclusions

Using a custom‐built Python algorithm, this study successfully combines both mono‐exponential and bi‐exponential models to analyze V̇O_2_ kinetics in the post‐exercise period. One of the key advantages of our approach is the ability to use user‐defined baseline V̇O_2_ values, offering a significant improvement over current estimation methods. Our results clearly show that the bi‐exponential model performs better than the mono‐exponential model in terms of *R* ^2^ values, providing valuable insights into the complex mechanisms of post‐exercise recovery. Importantly, our algorithm incorporates physiologically informed modeling, improving the accuracy of estimated parameters like amplitude and tau values. In conclusion, this study fills an important gap in the existing literature by offering a comprehensive and accurate way to analyze post‐exercise V̇O_2_ kinetics. Future research should aim to validate the model empirically, explore its applicability to different populations, and fine‐tune it for specialized use‐case. Overall, our work lays the groundwork for more personalized approaches to improve both athletic performance and recovery strategies.

## Author Contributions

Authors contributed to the concept and design (all authors), acquisition of the data (XX, XX, and XX), analysis (XX and XX) and interpretation (all authors), drafting and revision (all authors), final approval (all authors), and agreement to be accountable (all authors). The guarantor (XX) accepts full responsibility for the work and/or the conduct of the study, had access to the data, and controlled the decision to publish. The corresponding author attests that all listed authors meet the authorship criteria and that no others meeting the criteria have been omitted.

## Ethics Statement

The study received approval from the Ethics Committee of XXX University (Approval No: 11/04, Date: 28 September 2023), ensuring compliance with the ethical standards stipulated by the Declaration of Helsinki.

## Consent

Informed consent was obtained in written form from all participants prior to their involvement in the research, affirming their voluntary participation and comprehension of the study's procedures and objectives.

## Conflicts of Interest

The authors declare no conflicts of interest.

## Supporting information

Supporting Information S1

Supporting Information S2

Supporting Information S3

Supporting Information S4

Supporting Information S5

## Data Availability

The datasets generated and analyzed during the current study are included as Supporting Information accompanying this article (Appendix 2, Model parameters). The custom Python algorithm used for the analysis is available in a GitHub repository accessible at the following URL: https://github.com/XX/FitExpVO2_Modeling‐of‐Post‐exercise‐Oxygen‐Kinetic.
