# Supplementary material for: Enhancing Post‐Exercise Oxygen Kinetics Modeling With Physiological Bounds and Manual V̇O2_baseline Input: A Novel Approach
Source: Eur J Sport Sci. 2025 Apr 22;25(5):e12306. doi: 10.1002/ejsc.12306 (PMC12013733; doi:10.1002/ejsc.12306)
Supplement: Supplementary file 1 — Supporting Information S1 [file EJSC-25-e12306-s004.docx]

o Appendix 1: Time and oxygen data ("Impact of V̇O2_baseline variations on model parameters" section in the main document)

o Appendix 2: Model parameters ("Data availability" section in the title page document)

o Appendix 3: Model-fit graphs ("Comparative performance of models" section in the main document)

o Appendix 4: Python codes ("Software and Libraries" section in the main document)
