# Supplementary material for: Enhancing Post‐Exercise Oxygen Kinetics Modeling With Physiological Bounds and Manual V̇O2_baseline Input: A Novel Approach
Source: Eur J Sport Sci. 2025 Apr 22;25(5):e12306. doi: 10.1002/ejsc.12306 (PMC12013733; doi:10.1002/ejsc.12306)
Supplement: Supplementary file 4 — Supporting Information S4 [file EJSC-25-e12306-s005.pdf]

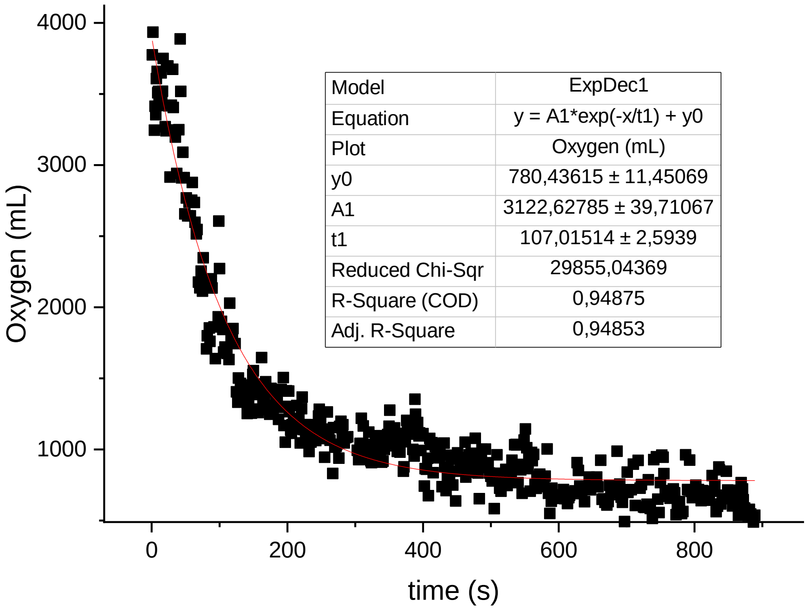

Figure 5. Application of Origin Software for Fitting the Mono-Exponential Model to Actual Data. *Note*: The presented data are based on the results from a participant serving as a representative example for illustration purposes (see Appendix 1, time1 and oxy1 data for participant 1).

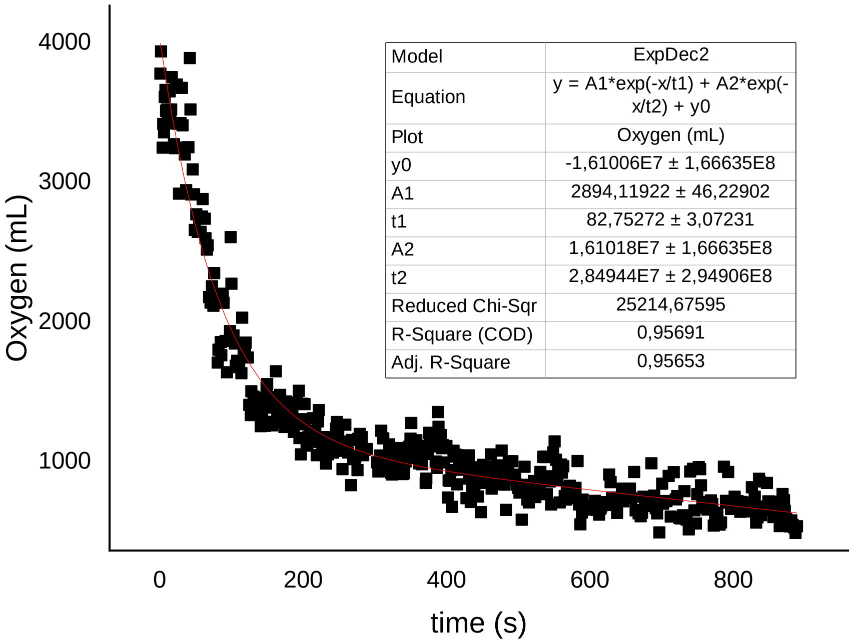

Figure 6. Application of Origin Software for Fitting the Bi-Exponential Model to Actual Data. *Note*: The presented data are based on the results from a participant serving as a representative example for illustration purposes (see Appendix 1, time1 and oxy1 data for participant 1).

Exponential fit data for the calculation of the Anaerobic Alactic System.

**v_0**: 781.17	Oxygen uptake at baseline
**A_1**: 1660.76	Amplitude - fast component
**A_2**: 1211.60	Amplitude - slow component
**τ_1**: 40.43	Time constant - fast component
**τ_2**: 171.81	Time constant - slow component
**R^2**: 0.49	Determination coefficient

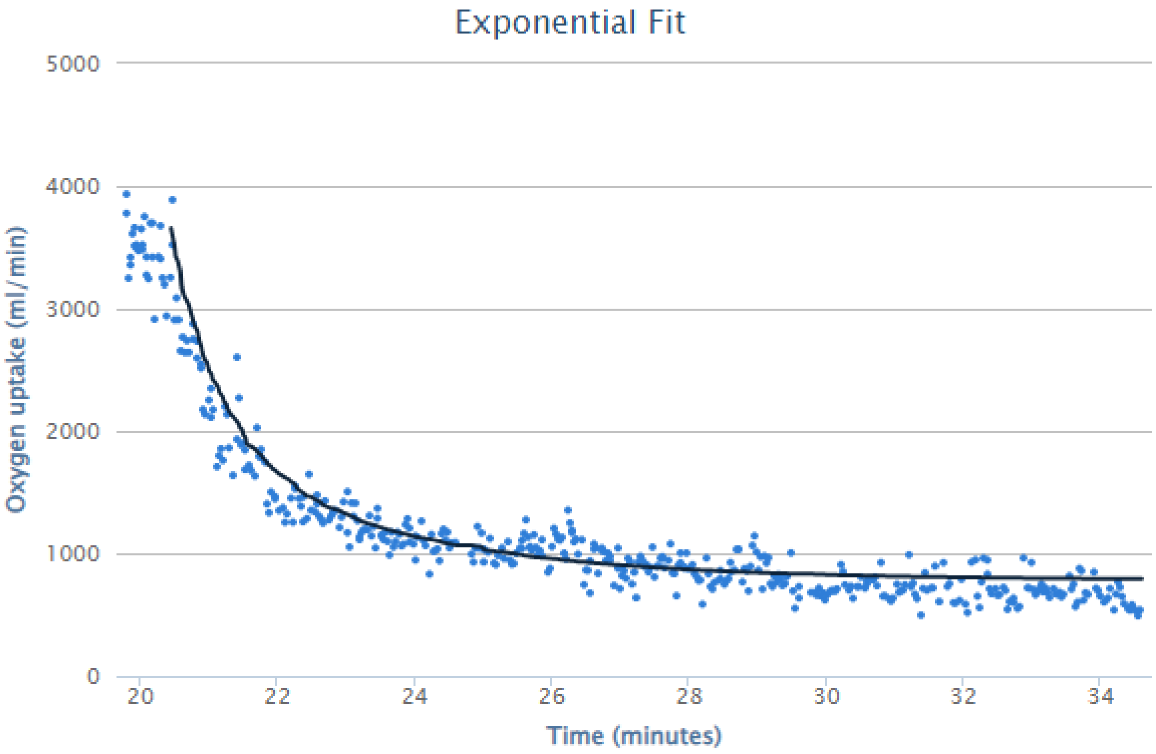

Figure 7. Application of GedaeLab Software for Fitting the Bi-Exponential Model to Actual Data. *Note*: The presented data are based on the results from a participant serving as a representative example for illustration purposes (see Appendix 1, time1 and oxy1 data for participant 1).

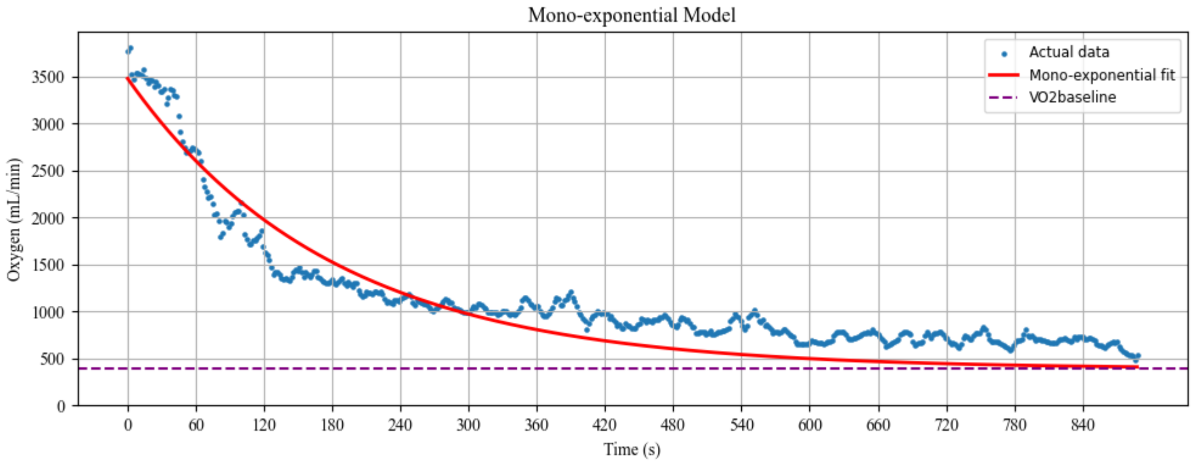

Figure 8. Application of Custom Python-Based Algorithm for Fitting the Mono-Exponential Model to Smoothed and Interpolated Actual Data. *Note*: The presented data are based on the results from a participant serving as a representative example for illustration purposes (see Appendix 1, time1 and oxy1 data for participant 1).

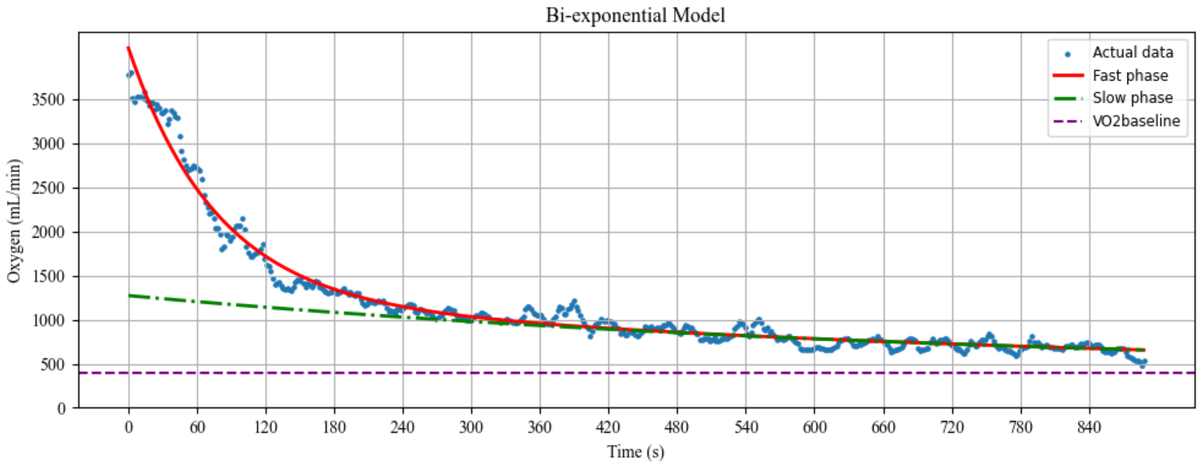

Figure 9. Application of Custom Python-Based Algorithm for Fitting the Bi-Exponential Model to Smoothed and Interpolated Actual Data. *Note*: The presented data are based on the results from a participant serving as a representative example for illustration purposes (see Appendix 1, time1 and oxy1 data for participant 1).

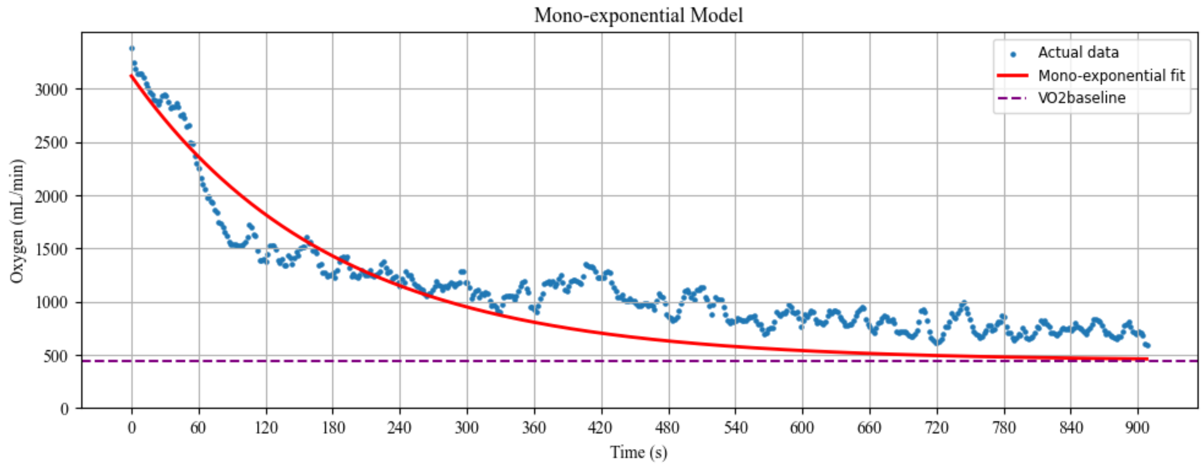

Figure 10. Application of Custom Python-Based Algorithm for Fitting the Mono-Exponential Model to Smoothed and Interpolated Actual Data. *Note*: The presented data are based on the results from a participant serving as a representative example for illustration purposes (see Appendix 1, time2 and oxy2 data for participant 2).

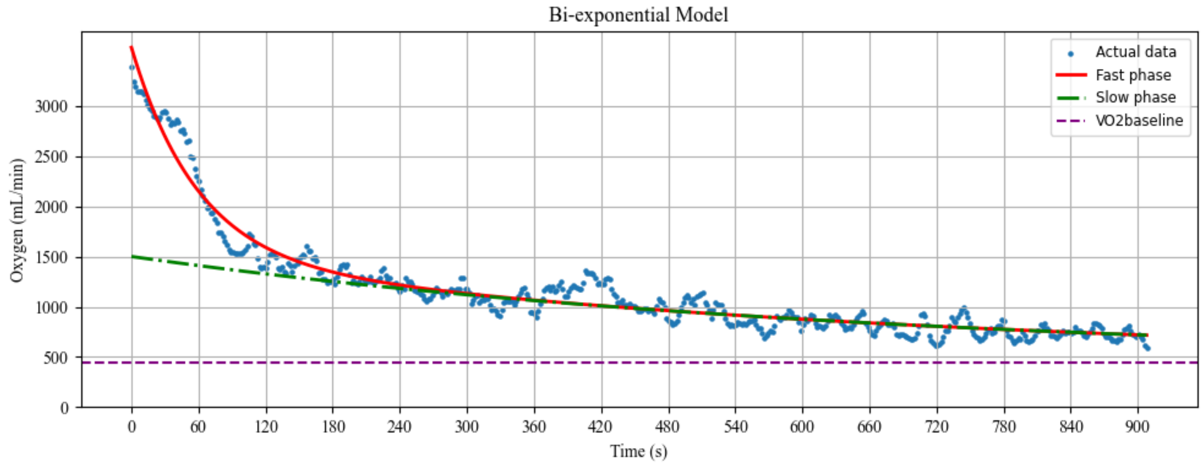

Figure 11. Application of Custom Python-Based Algorithm for Fitting the Bi-Exponential Model to Smoothed and Interpolated Actual Data. *Note*: The presented data are based on the results from a participant serving as a representative example for illustration purposes (see Appendix 1, time2 and oxy2 data for participant 2).

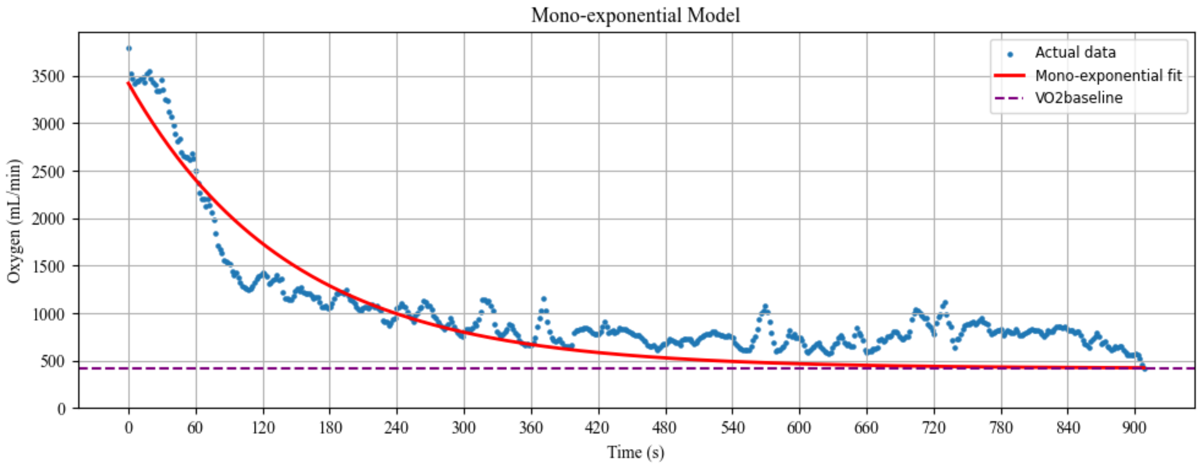

Figure 12. Application of Custom Python-Based Algorithm for Fitting the Mono-Exponential Model to Smoothed and Interpolated Actual Data. *Note*: The presented data are based on the results from a participant serving as a representative example for illustration purposes (see Appendix 1, time3 and oxy3 data for participant 3).

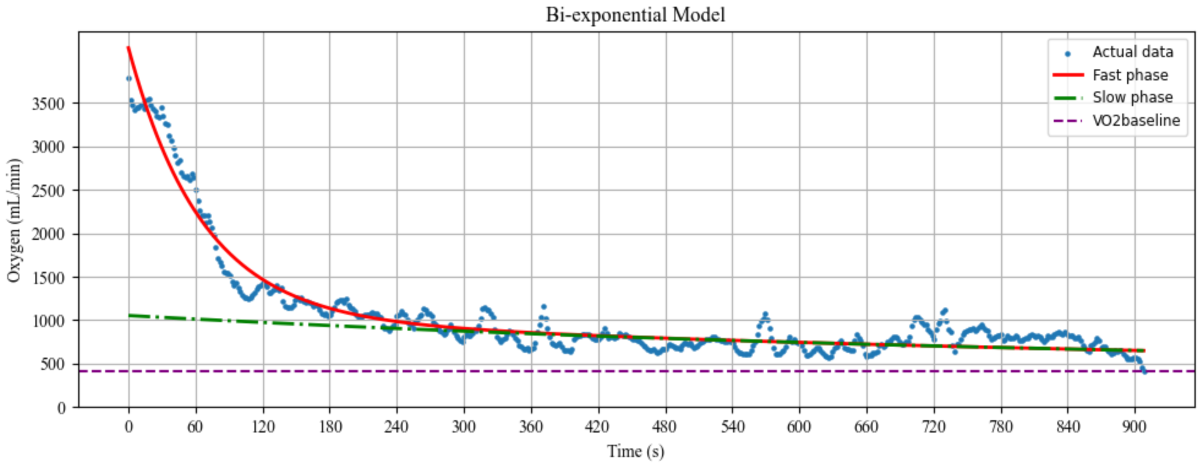

Figure 13. Application of Custom Python-Based Algorithm for Fitting the Bi-Exponential Model to Smoothed and Interpolated Actual Data. *Note*: The presented data are based on the results from a participant serving as a representative example for illustration purposes (see Appendix 1, time3 and oxy3 data for participant 3).

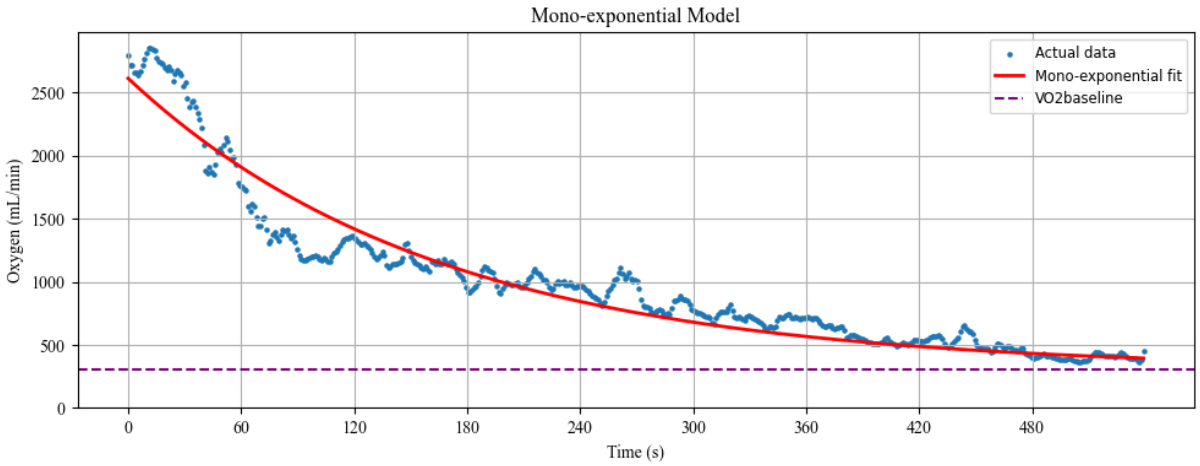

Figure 14. Application of Custom Python-Based Algorithm for Fitting the Mono-Exponential Model to Smoothed and Interpolated Actual Data. *Note*: The presented data are based on the results from a participant serving as a representative example for illustration purposes (see Appendix 1, time4 and oxy4 data for participant 4).

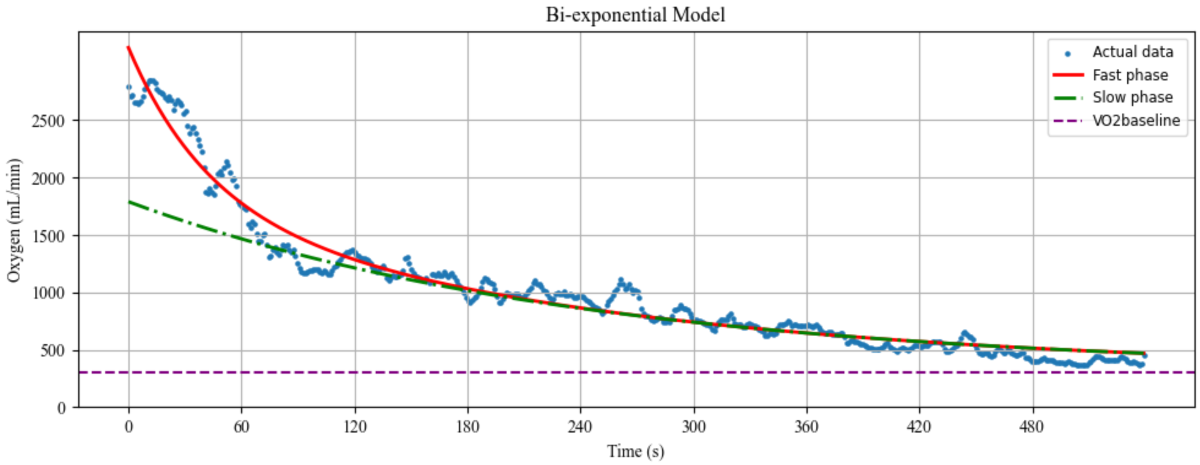

Figure 15. Application of Custom Python-Based Algorithm for Fitting the Bi-Exponential Model to Smoothed and Interpolated Actual Data. *Note*: The presented data are based on the results from a participant serving as a representative example for illustration purposes (see Appendix 1, time4 and oxy4 data for participant 4).

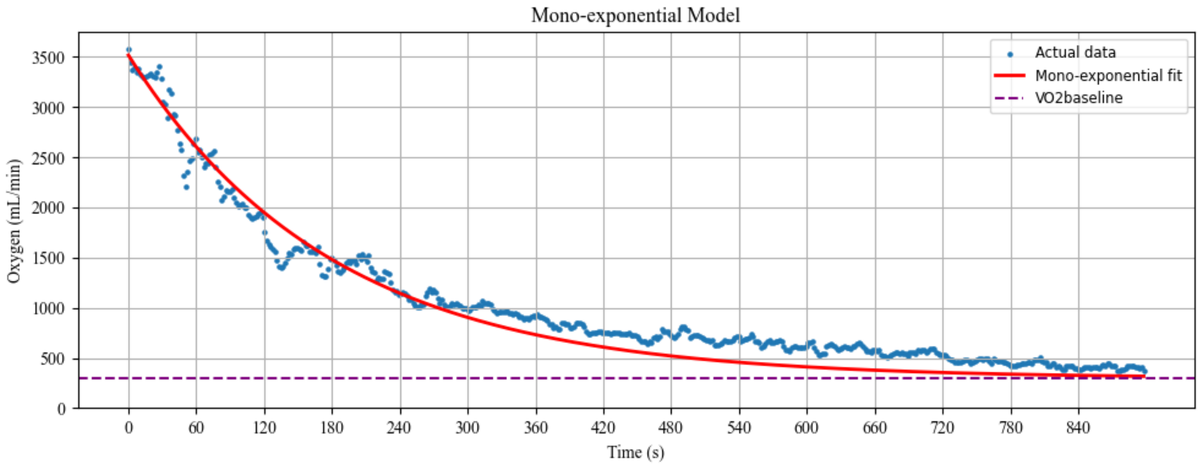

Figure 16. Application of Custom Python-Based Algorithm for Fitting the Mono-Exponential Model to Smoothed and Interpolated Actual Data. *Note*: The presented data are based on the results from a participant serving as a representative example for illustration purposes (see Appendix 1, time5 and oxy5 data for participant 5).

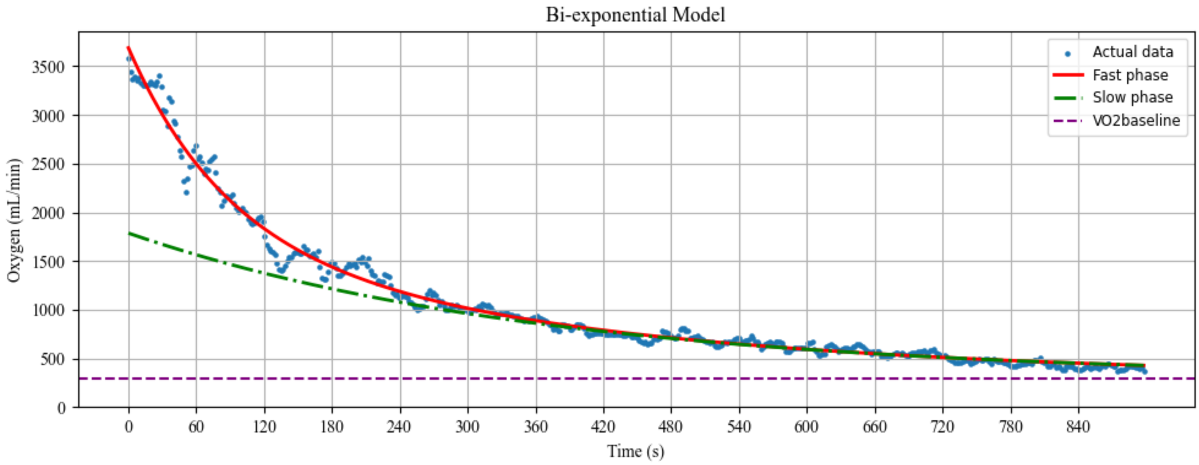

Figure 17. Application of Custom Python-Based Algorithm for Fitting the Bi-Exponential Model to Smoothed and Interpolated Actual Data. *Note*: The presented data are based on the results from a participant serving as a representative example for illustration purposes (see Appendix 1, time5 and oxy5 data for participant 5).

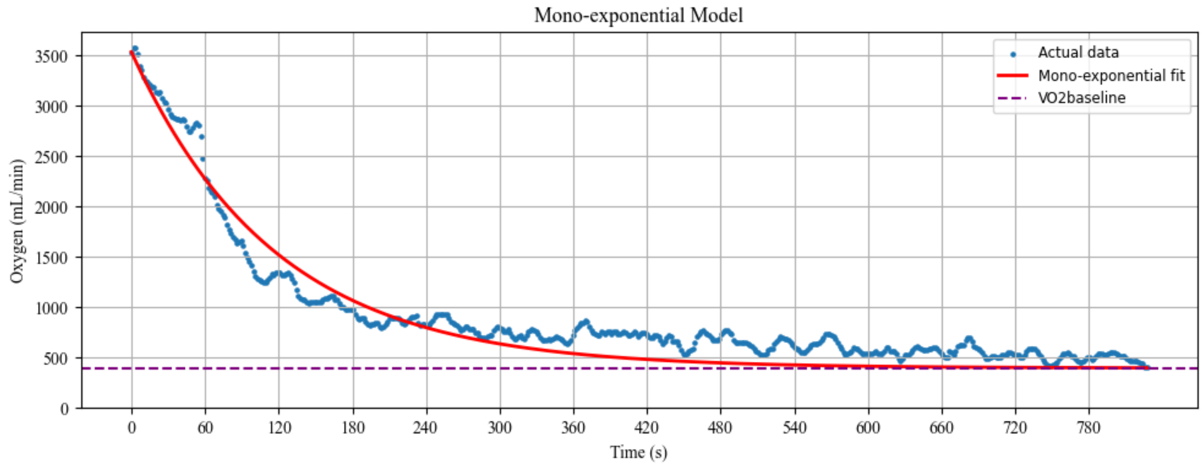

Figure 18. Application of Custom Python-Based Algorithm for Fitting the Mono-Exponential Model to Smoothed and Interpolated Actual Data. *Note*: The presented data are based on the results from a participant serving as a representative example for illustration purposes (see Appendix 1, time6 and oxy6 data for participant 6).

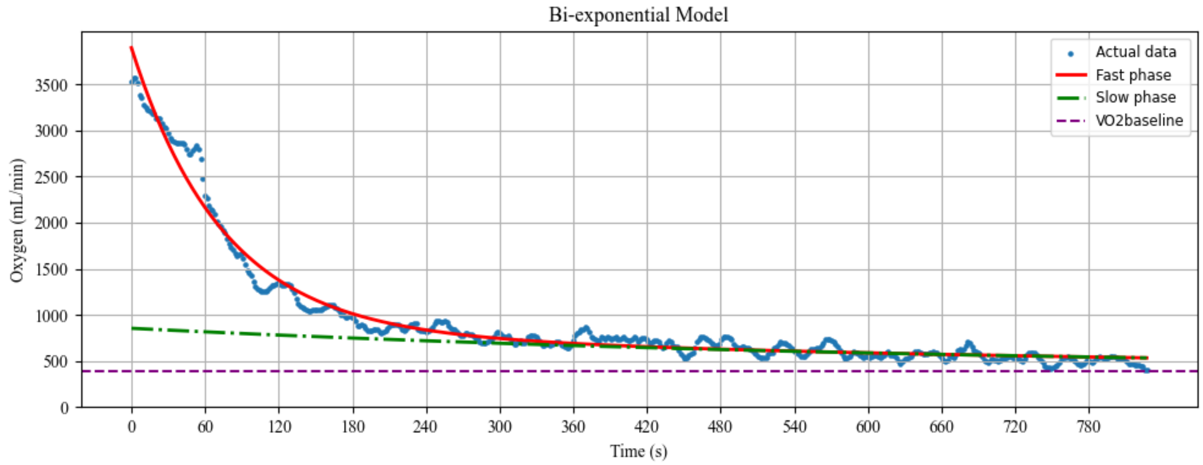

Figure 19. Application of Custom Python-Based Algorithm for Fitting the Bi-Exponential Model to Smoothed and Interpolated Actual Data. *Note*: The presented data are based on the results from a participant serving as a representative example for illustration purposes (see Appendix 1, time6 and oxy6 data for participant 6).

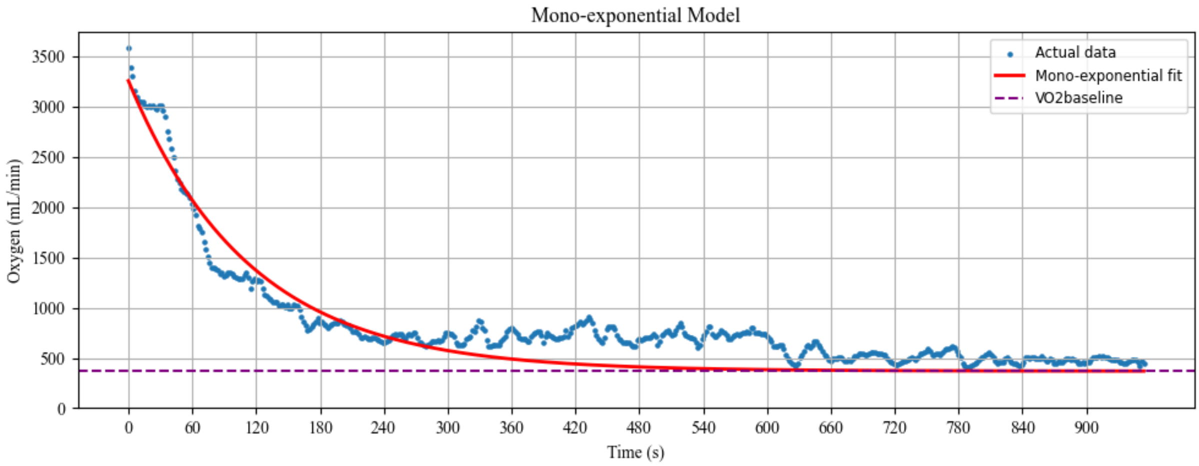

Figure 20. Application of Custom Python-Based Algorithm for Fitting the Mono-Exponential Model to Smoothed and Interpolated Actual Data. *Note*: The presented data are based on the results from a participant serving as a representative example for illustration purposes (see Appendix 1, time7 and oxy7 data for participant 7).

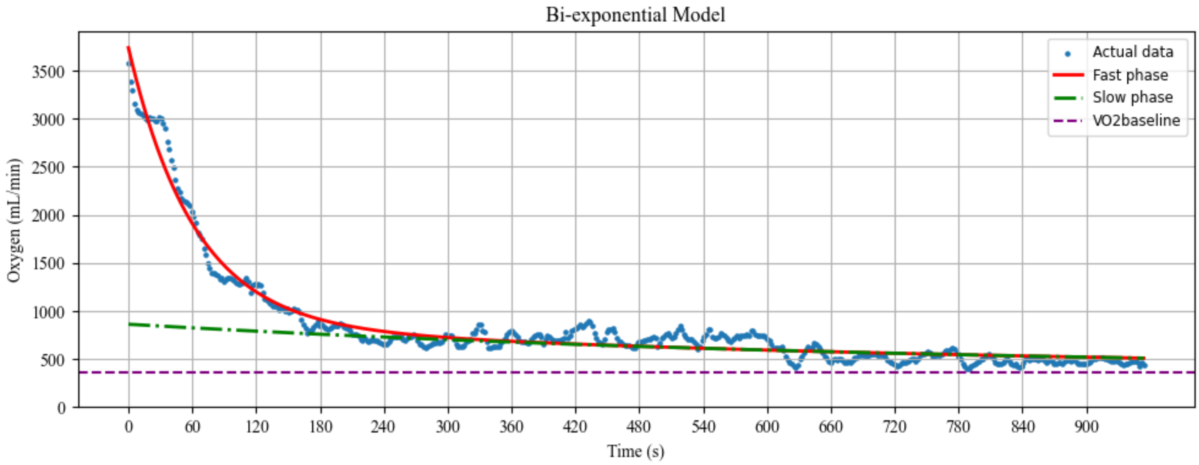

Figure 21. Application of Custom Python-Based Algorithm for Fitting the Bi-Exponential Model to Smoothed and Interpolated Actual Data. *Note*: The presented data are based on the results from a participant serving as a representative example for illustration purposes (see Appendix 1, time7 and oxy7 data for participant 7).

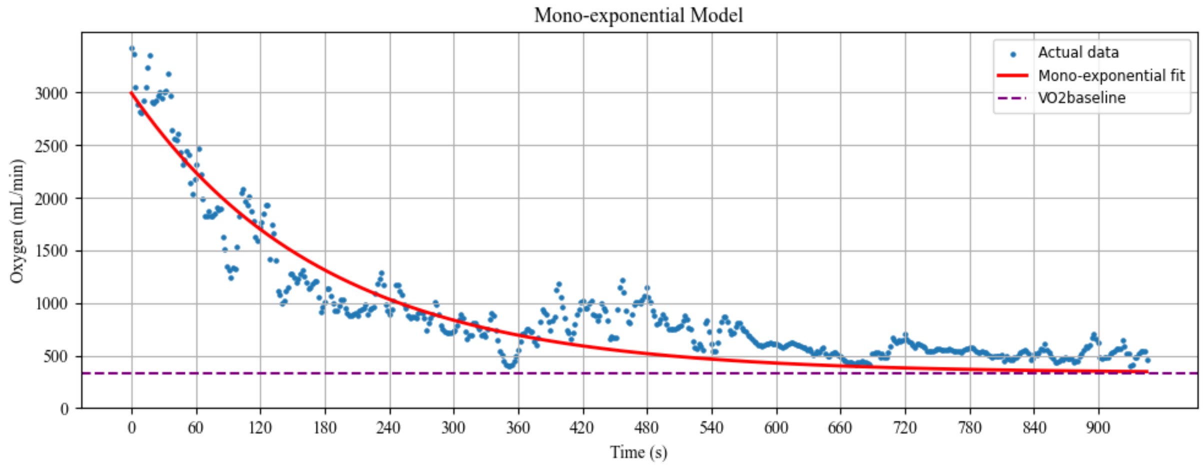

Figure 22. Application of Custom Python-Based Algorithm for Fitting the Mono-Exponential Model to Smoothed and Interpolated Actual Data. *Note*: The presented data are based on the results from a participant serving as a representative example for illustration purposes (see Appendix 1, time8 and oxy8 data for participant 8).

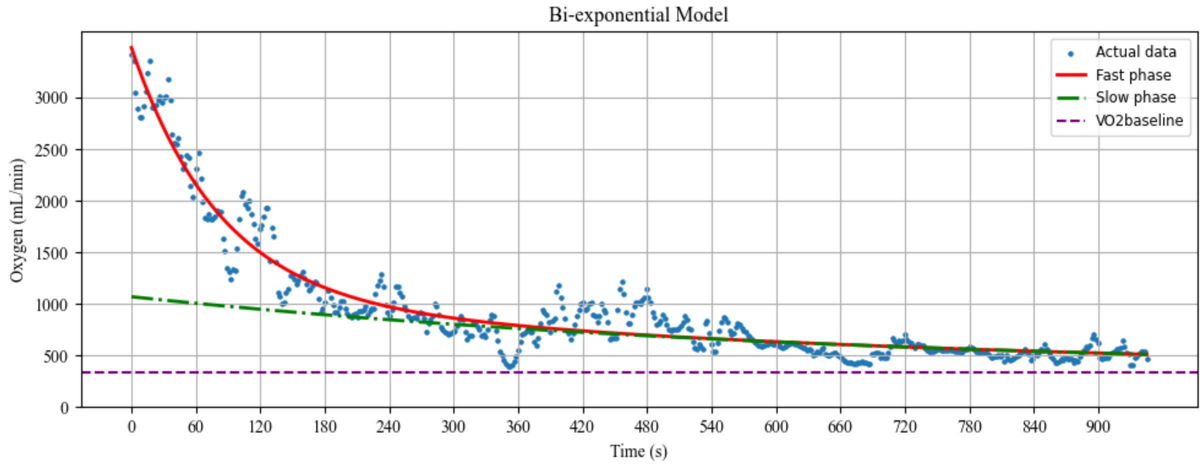

Figure 23. Application of Custom Python-Based Algorithm for Fitting the Bi-Exponential Model to Smoothed and Interpolated Actual Data. *Note*: The presented data are based on the results from a participant serving as a representative example for illustration purposes (see Appendix 1, time8 and oxy8 data for participant 8).

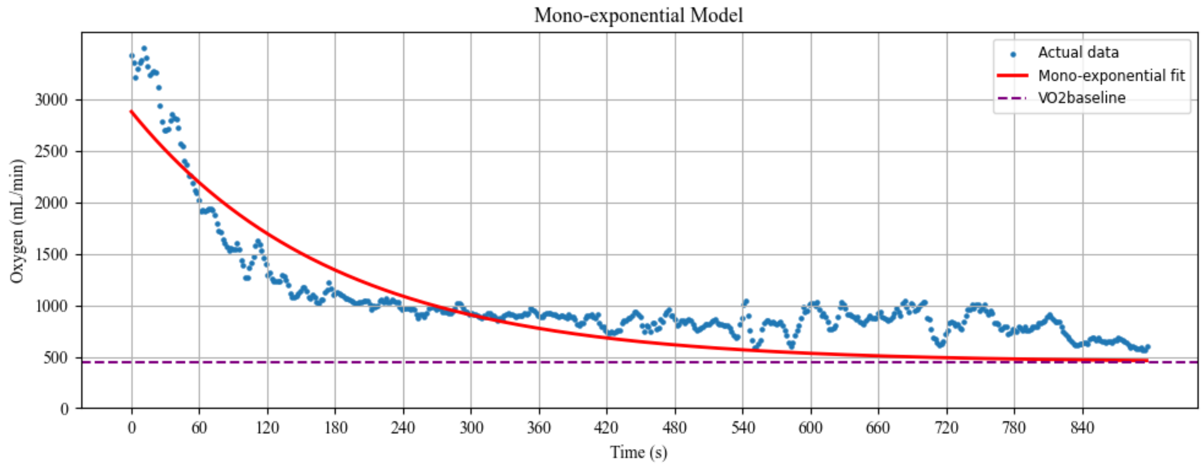

Figure 24. Application of Custom Python-Based Algorithm for Fitting the Mono-Exponential Model to Smoothed and Interpolated Actual Data. *Note*: The presented data are based on the results from a participant serving as a representative example for illustration purposes (see Appendix 1, time9 and oxy9 data for participant 9).

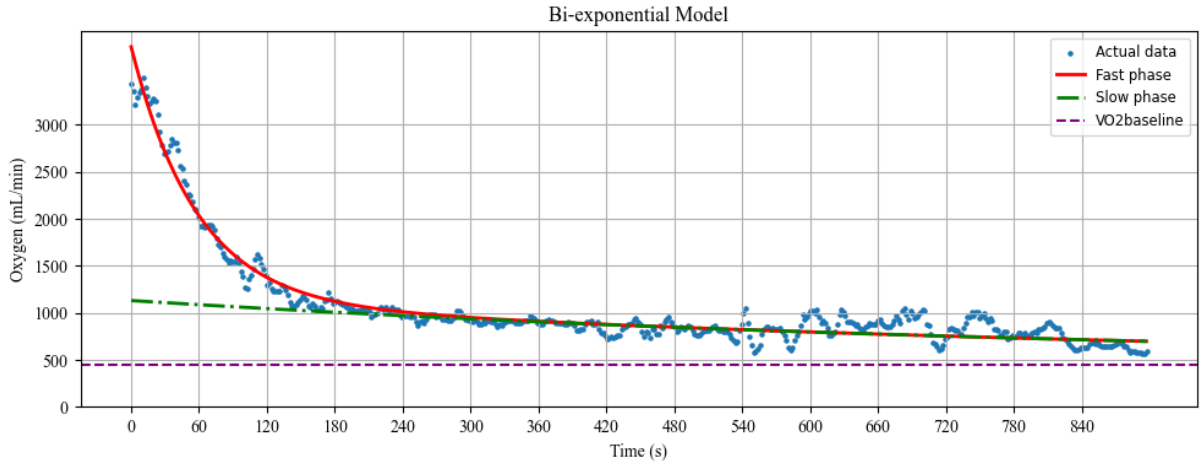

Figure 25. Application of Custom Python-Based Algorithm for Fitting the Bi-Exponential Model to Smoothed and Interpolated Actual Data. *Note*: The presented data are based on the results from a participant serving as a representative example for illustration purposes (see Appendix 1, time9 and oxy9 data for participant 9).

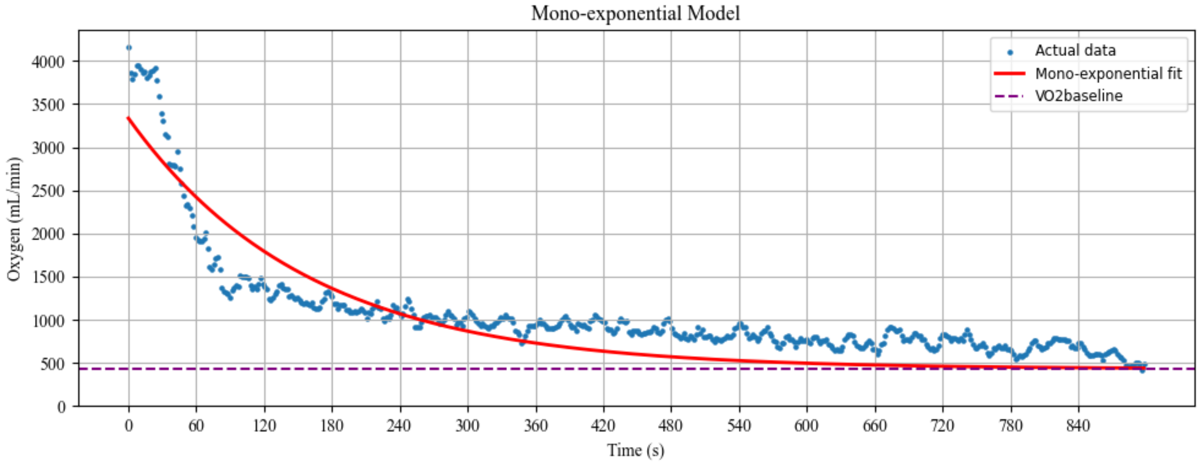

Figure 26. Application of Custom Python-Based Algorithm for Fitting the Mono-Exponential Model to Smoothed and Interpolated Actual Data. *Note*: The presented data are based on the results from a participant serving as a representative example for illustration purposes (see Appendix 1, time10 and oxy10 data for participant 10).

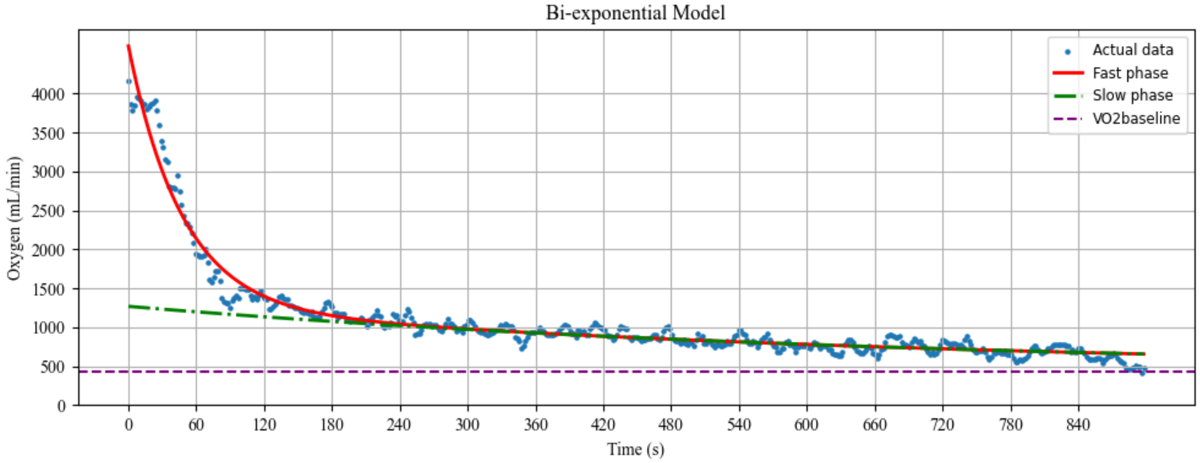

Figure 27. Application of Custom Python-Based Algorithm for Fitting the Bi-Exponential Model to Smoothed and Interpolated Actual Data. *Note*: The presented data are based on the results from a participant serving as a representative example for illustration purposes (see Appendix 1, time10 and oxy10 data for participant 10).

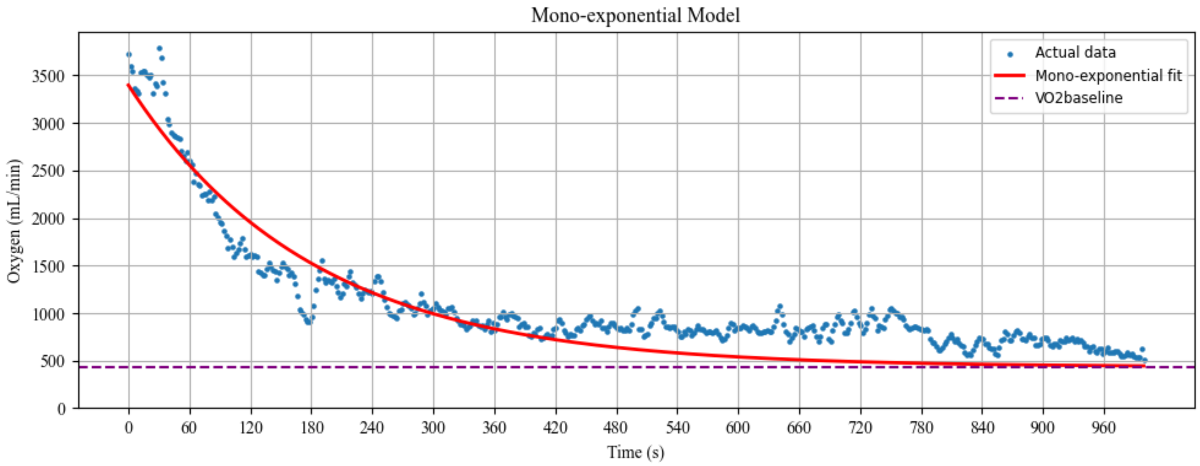

Figure 28. Application of Custom Python-Based Algorithm for Fitting the Mono-Exponential Model to Smoothed and Interpolated Actual Data. *Note*: The presented data are based on the results from a participant serving as a representative example for illustration purposes (see Appendix 1, time11 and oxy11 data for participant 11).

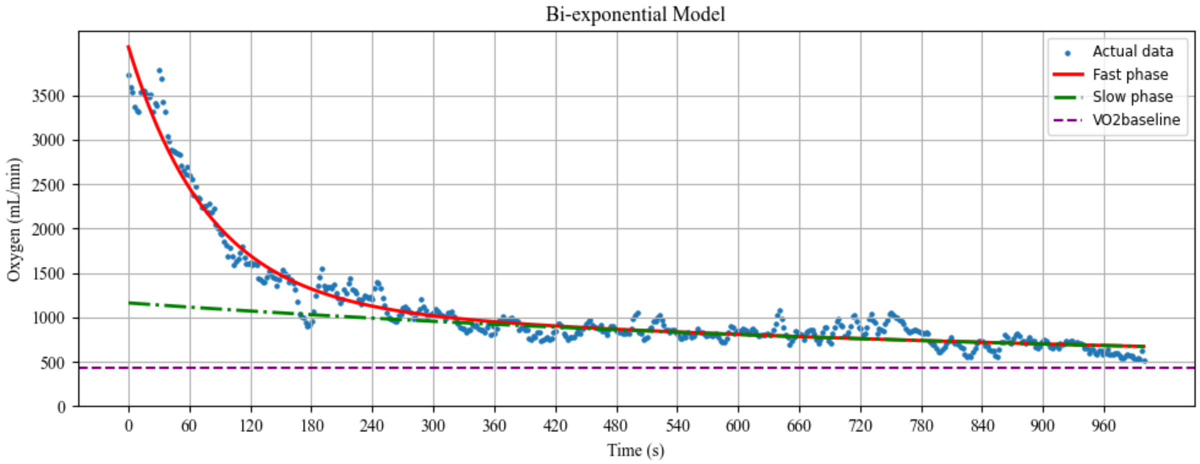

Figure 29. Application of Custom Python-Based Algorithm for Fitting the Bi-Exponential Model to Smoothed and Interpolated Actual Data. *Note*: The presented data are based on the results from a participant serving as a representative example for illustration purposes (see Appendix 1, time11 and oxy11 data for participant 11).

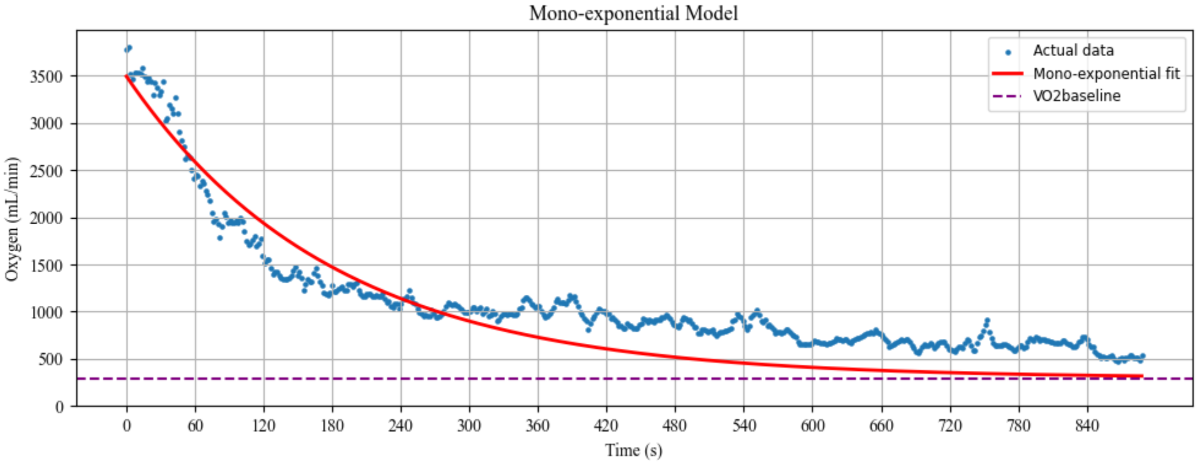

Figure 30. Application of Custom Python-Based Algorithm for Fitting the Mono-Exponential Model to Smoothed and Interpolated Actual Data. *Note*: The presented data are based on the results from a participant serving as a representative example for illustration purposes (see Appendix 1, time12 and oxy12 data for participant 12).

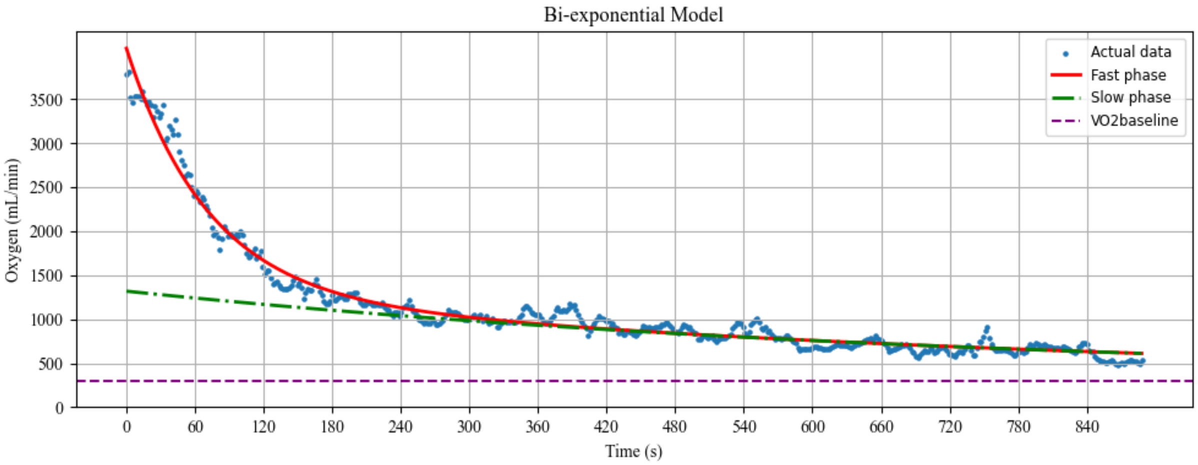

Figure 31. Application of Custom Python-Based Algorithm for Fitting the Bi-Exponential Model to Smoothed and Interpolated Actual Data. *Note*: The presented data are based on the results from a participant serving as a representative example for illustration purposes (see Appendix 1, time12 and oxy12 data for participant 12).

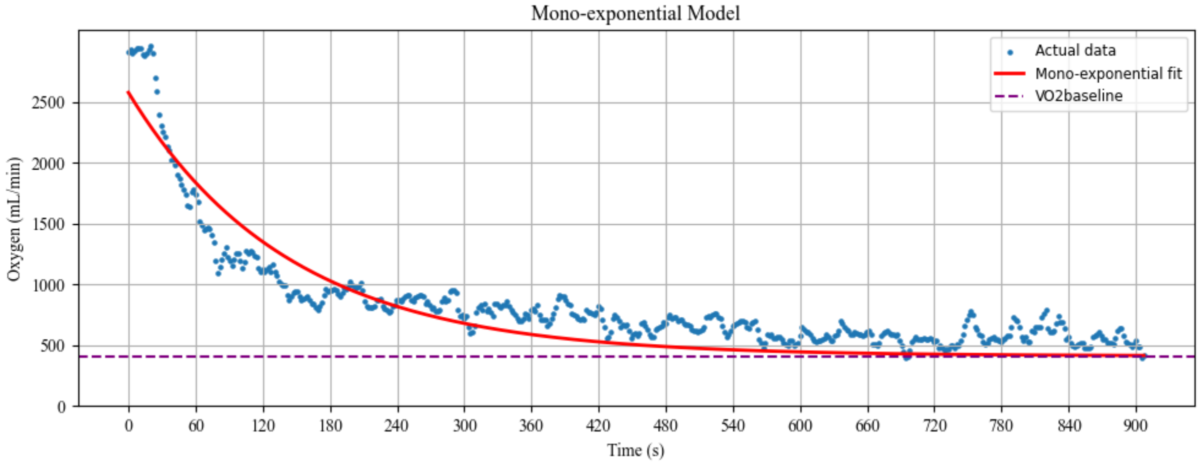

Figure 32. Application of Custom Python-Based Algorithm for Fitting the Mono-Exponential Model to Smoothed and Interpolated Actual Data. *Note*: The presented data are based on the results from a participant serving as a representative example for illustration purposes (see Appendix 1, time13 and oxy13 data for participant 13).

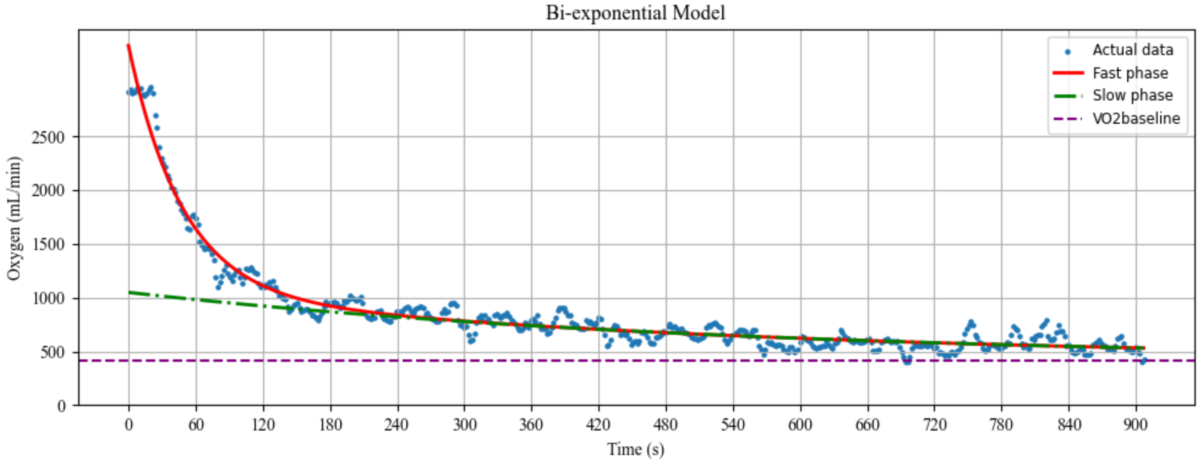

Figure 33. Application of Custom Python-Based Algorithm for Fitting the Bi-Exponential Model to Smoothed and Interpolated Actual Data. *Note*: The presented data are based on the results from a participant serving as a representative example for illustration purposes (see Appendix 1, time13 and oxy13 data for participant 13).

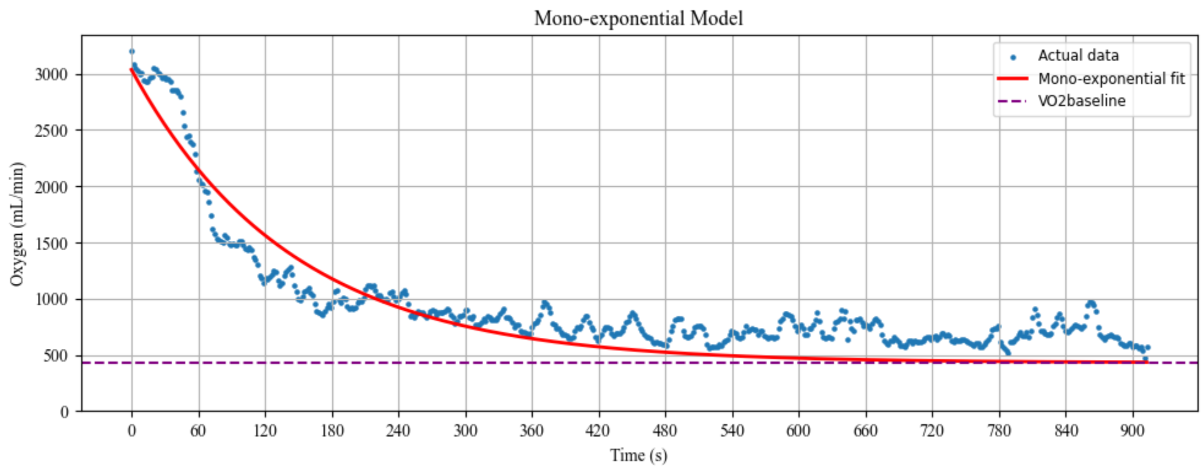

Figure 34. Application of Custom Python-Based Algorithm for Fitting the Mono-Exponential Model to Smoothed and Interpolated Actual Data. *Note*: The presented data are based on the results from a participant serving as a representative example for illustration purposes (see Appendix 1, time14 and oxy14 data for participant 14).

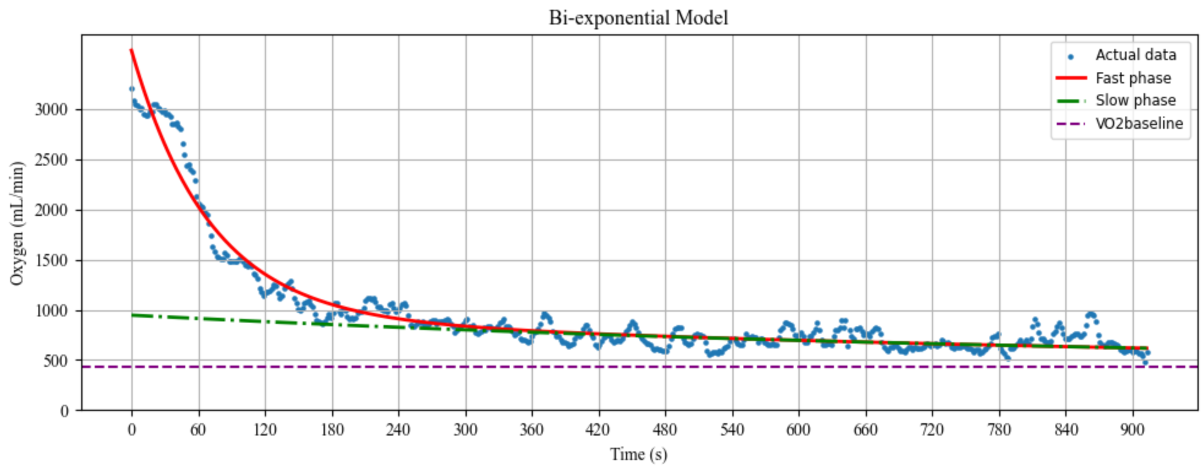

Figure 35. Application of Custom Python-Based Algorithm for Fitting the Bi-Exponential Model to Smoothed and Interpolated Actual Data. *Note*: The presented data are based on the results from a participant serving as a representative example for illustration purposes (see Appendix 1, time14 and oxy14 data for participant 14).

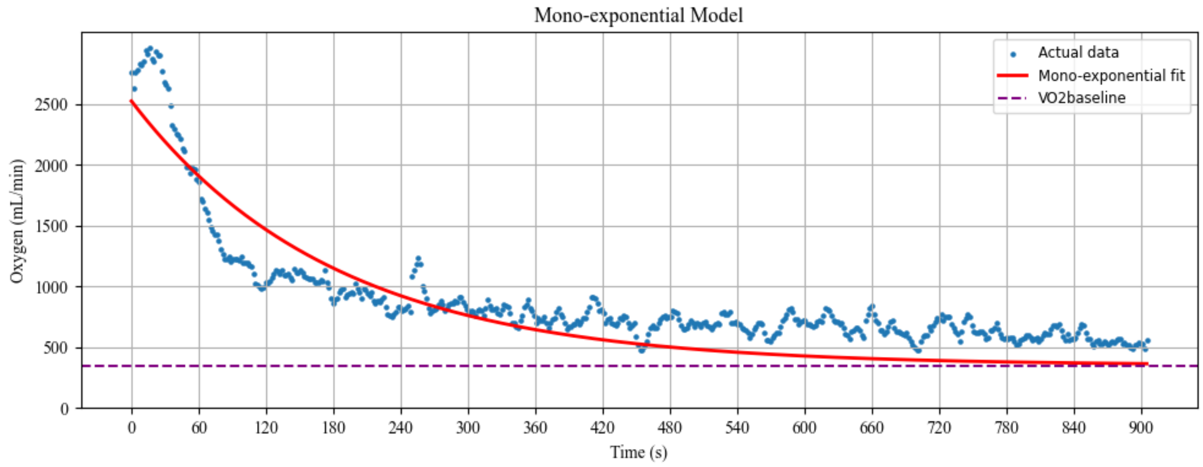

Figure 36. Application of Custom Python-Based Algorithm for Fitting the Mono-Exponential Model to Smoothed and Interpolated Actual Data. *Note*: The presented data are based on the results from a participant serving as a representative example for illustration purposes (see Appendix 1, time15 and oxy15 data for participant 15).

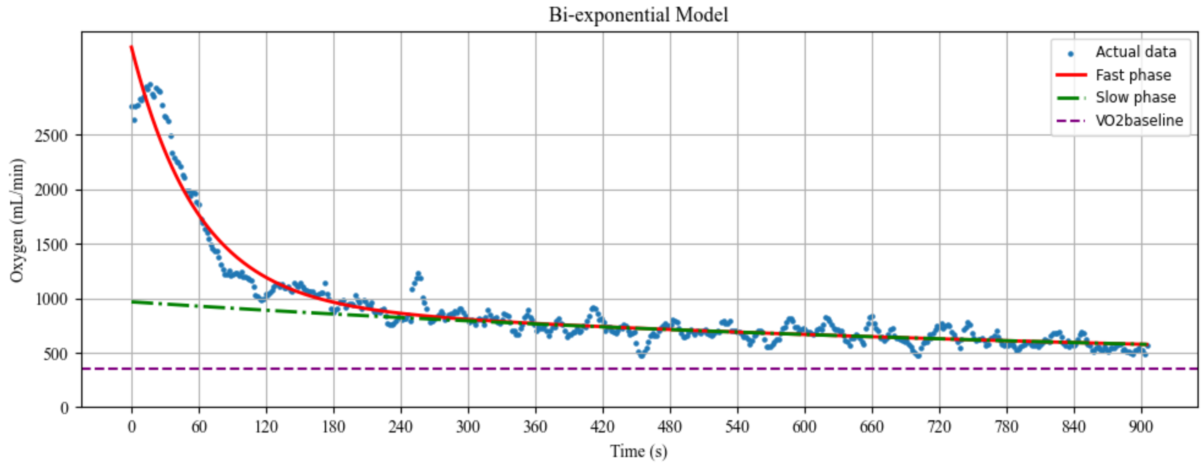

Figure 37. Application of Custom Python-Based Algorithm for Fitting the Bi-Exponential Model to Smoothed and Interpolated Actual Data. *Note*: The presented data are based on the results from a participant serving as a representative example for illustration purposes (see Appendix 1, time15 and oxy15 data for participant 15).

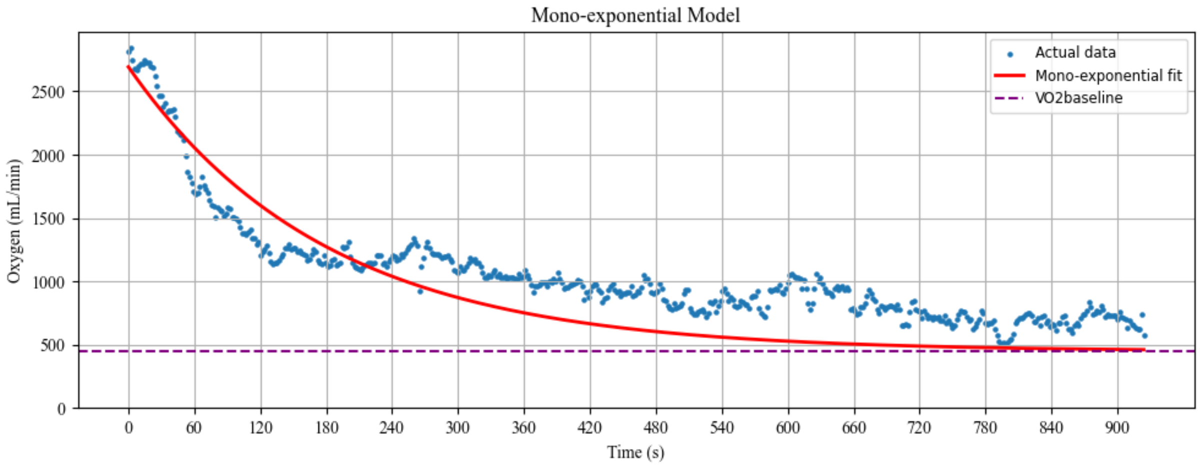

Figure 38. Application of Custom Python-Based Algorithm for Fitting the Mono-Exponential Model to Smoothed and Interpolated Actual Data. *Note*: The presented data are based on the results from a participant serving as a representative example for illustration purposes (see Appendix 1, time16 and oxy16 data for participant 16).

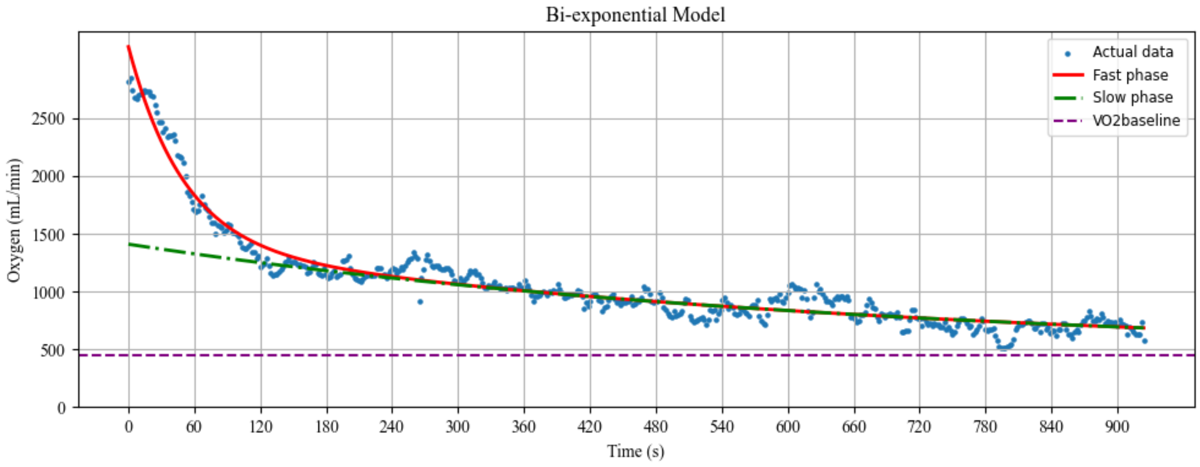

Figure 39. Application of Custom Python-Based Algorithm for Fitting the Bi-Exponential Model to Smoothed and Interpolated Actual Data. *Note*: The presented data are based on the results from a participant serving as a representative example for illustration purposes (see Appendix 1, time16 and oxy16 data for participant 16).

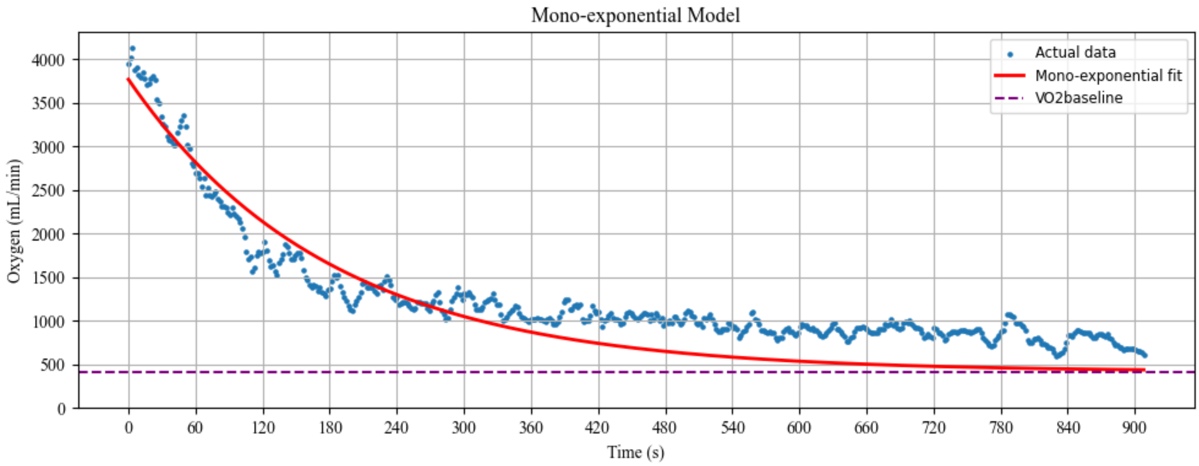

Figure 40. Application of Custom Python-Based Algorithm for Fitting the Mono-Exponential Model to Smoothed and Interpolated Actual Data. *Note*: The presented data are based on the results from a participant serving as a representative example for illustration purposes (see Appendix 1, time17 and oxy17 data for participant 17).

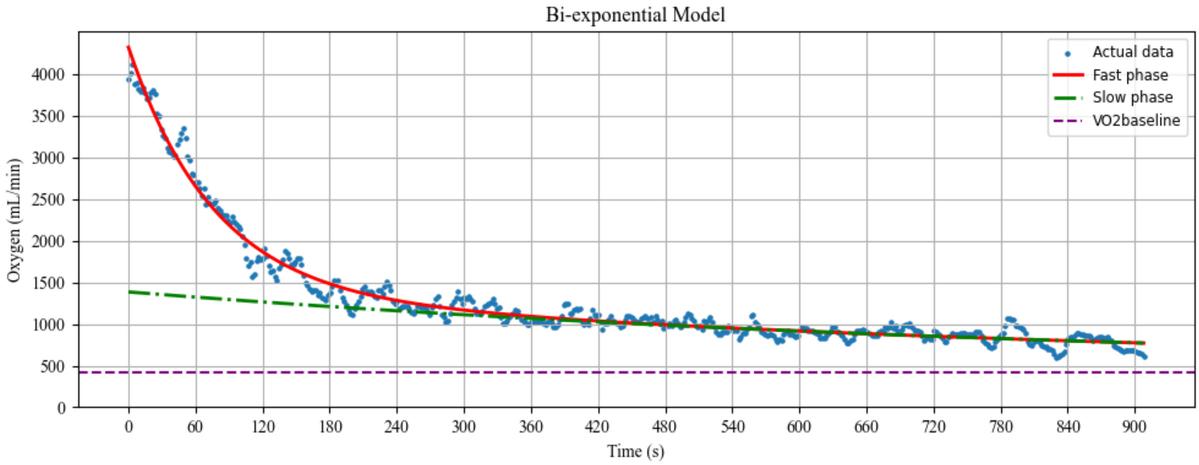

Figure 41. Application of Custom Python-Based Algorithm for Fitting the Bi-Exponential Model to Smoothed and Interpolated Actual Data. *Note*: The presented data are based on the results from a participant serving as a representative example for illustration purposes (see Appendix 1, time17 and oxy17 data for participant 17).

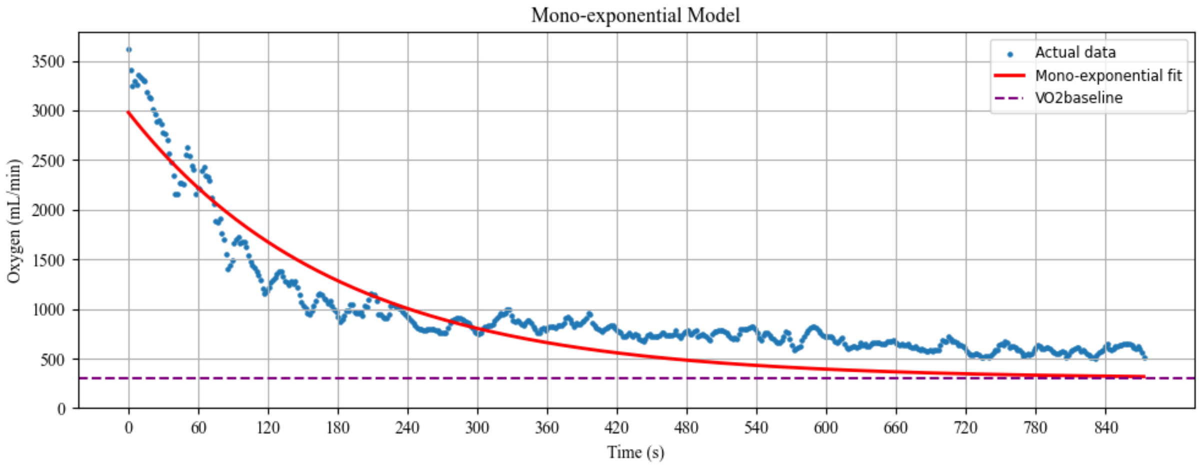

Figure 42. Application of Custom Python-Based Algorithm for Fitting the Mono-Exponential Model to Smoothed and Interpolated Actual Data. *Note*: The presented data are based on the results from a participant serving as a representative example for illustration purposes (see Appendix 1, time18 and oxy18 data for participant 18).

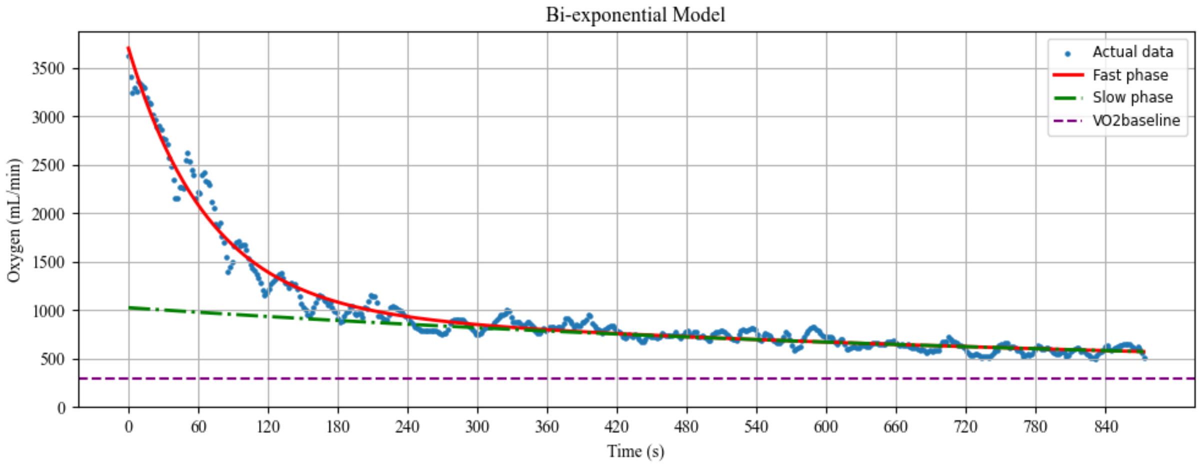

Figure 43. Application of Custom Python-Based Algorithm for Fitting the Bi-Exponential Model to Smoothed and Interpolated Actual Data. *Note*: The presented data are based on the results from a participant serving as a representative example for illustration purposes (see Appendix 1, time18 and oxy18 data for participant 18).

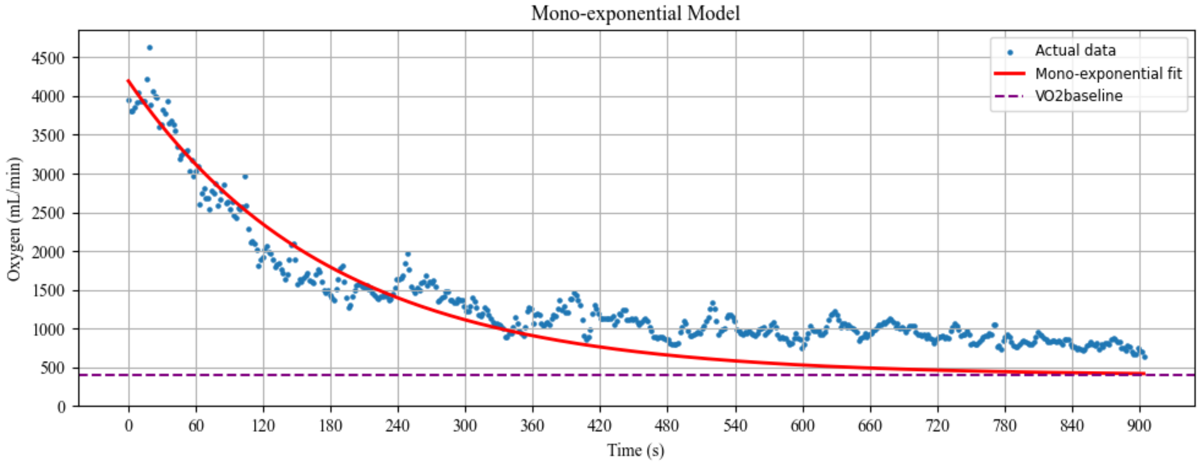

Figure 44. Application of Custom Python-Based Algorithm for Fitting the Mono-Exponential Model to Smoothed and Interpolated Actual Data. *Note*: The presented data are based on the results from a participant serving as a representative example for illustration purposes (see Appendix 1, time19 and oxy19 data for participant 19).

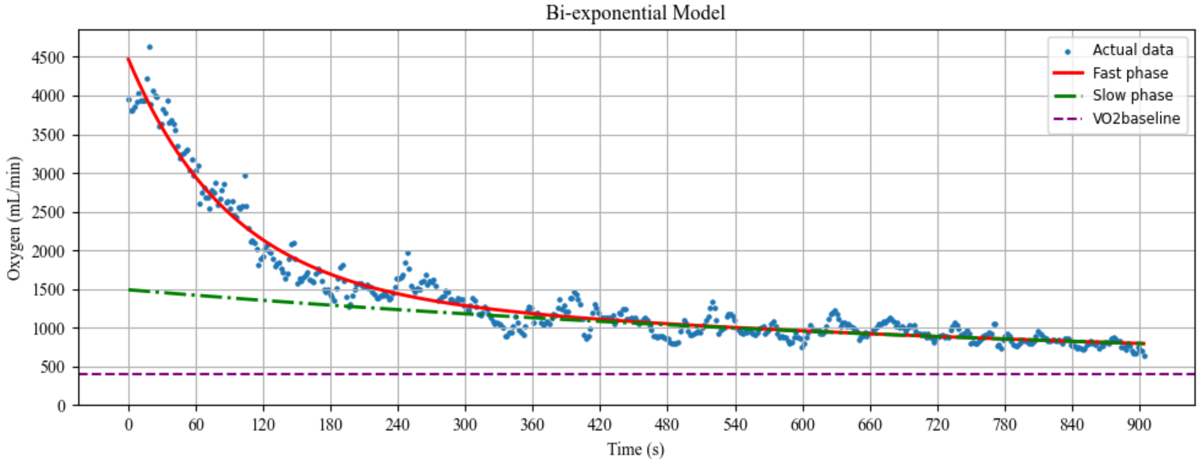

Figure 45. Application of Custom Python-Based Algorithm for Fitting the Bi-Exponential Model to Smoothed and Interpolated Actual Data. *Note*: The presented data are based on the results from a participant serving as a representative example for illustration purposes (see Appendix 1, time19 and oxy19 data for participant 19).

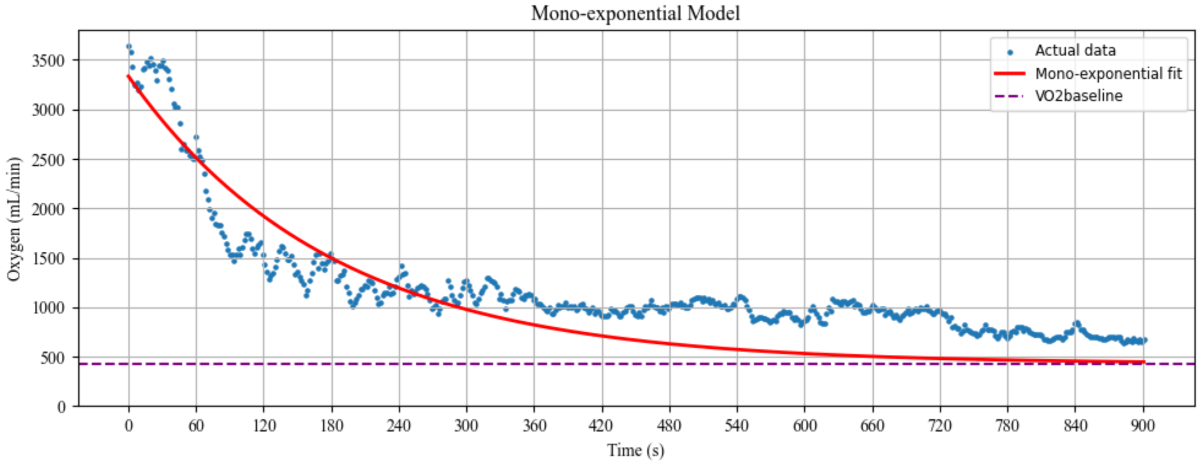

Figure 46. Application of Custom Python-Based Algorithm for Fitting the Mono-Exponential Model to Smoothed and Interpolated Actual Data. *Note*: The presented data are based on the results from a participant serving as a representative example for illustration purposes (see Appendix 1, time20 and oxy20 data for participant 20).

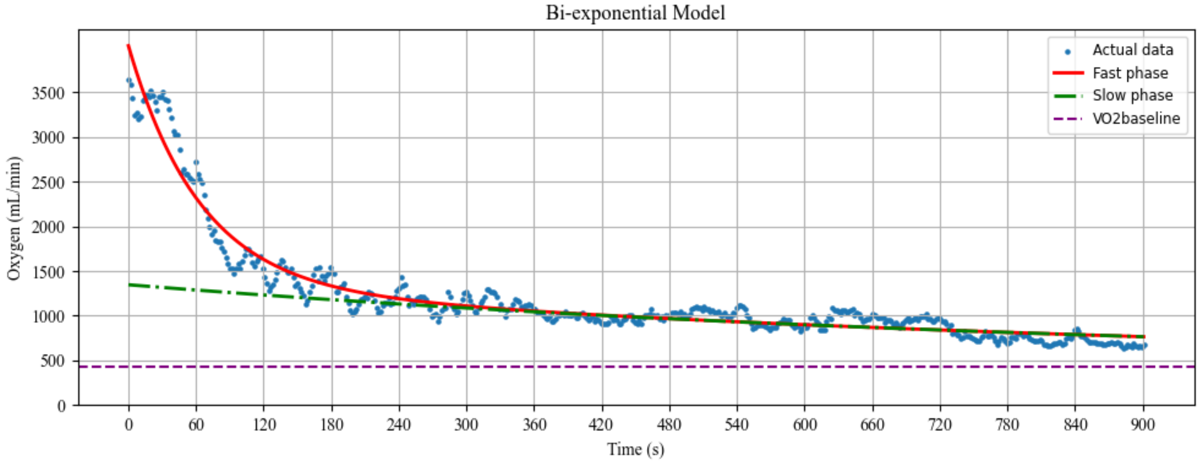

Figure 47. Application of Custom Python-Based Algorithm for Fitting the Bi-Exponential Model to Smoothed and Interpolated Actual Data. *Note*: The presented data are based on the results from a participant serving as a representative example for illustration purposes (see Appendix 1, time20 and oxy20 data for participant 20).

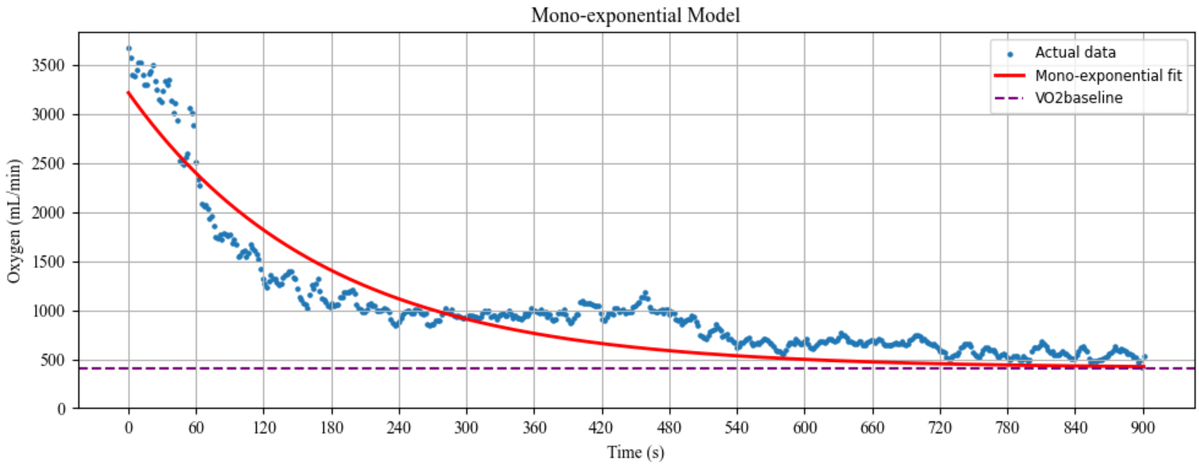

Figure 48. Application of Custom Python-Based Algorithm for Fitting the Mono-Exponential Model to Smoothed and Interpolated Actual Data. *Note*: The presented data are based on the results from a participant serving as a representative example for illustration purposes (see Appendix 1, time21 and oxy21 data for participant 21).

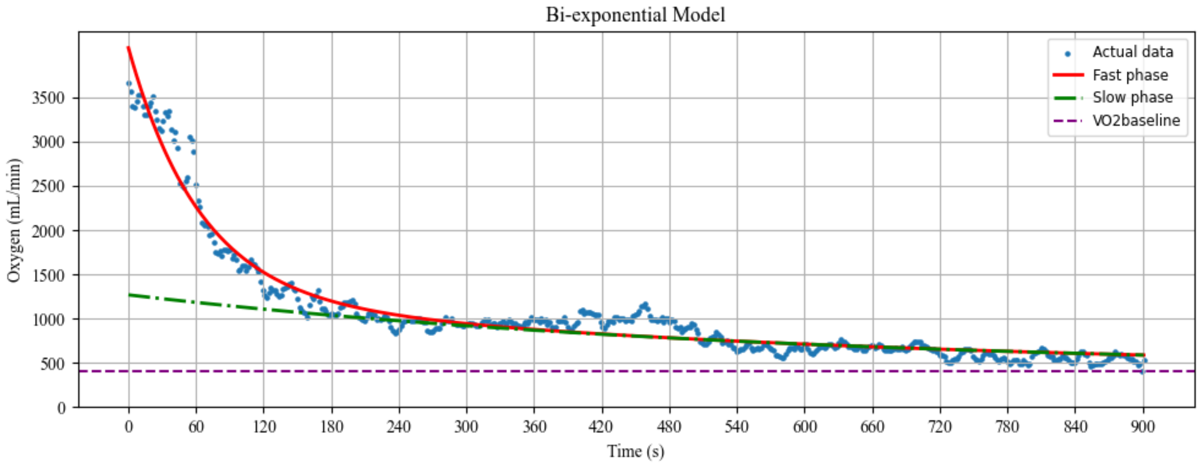

Figure 49. Application of Custom Python-Based Algorithm for Fitting the Bi-Exponential Model to Smoothed and Interpolated Actual Data. *Note*: The presented data are based on the results from a participant serving as a representative example for illustration purposes (see Appendix 1, time21 and oxy21 data for participant 21).

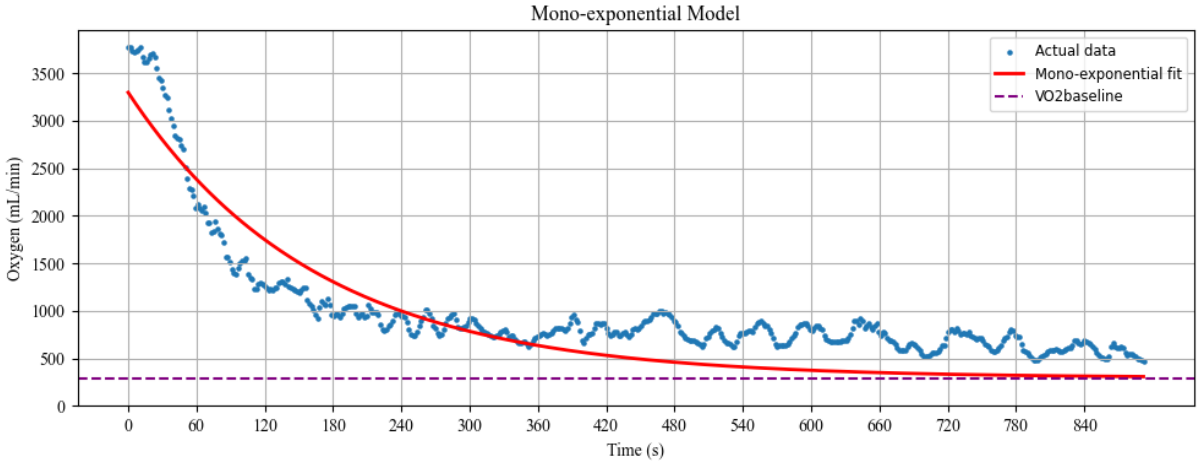

Figure 50. Application of Custom Python-Based Algorithm for Fitting the Mono-Exponential Model to Smoothed and Interpolated Actual Data. *Note*: The presented data are based on the results from a participant serving as a representative example for illustration purposes (see Appendix 1, time22 and oxy22 data for participant 22).

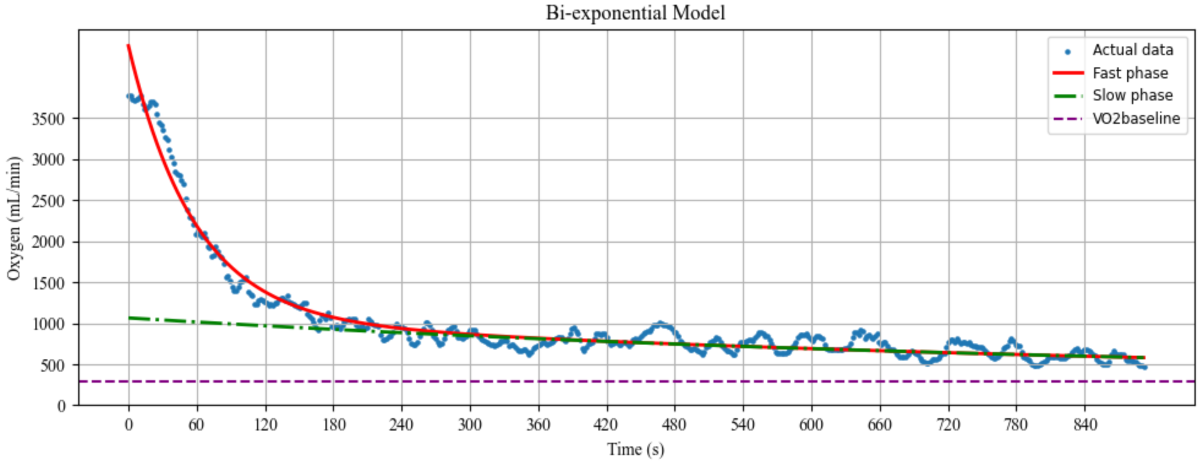

Figure 51. Application of Custom Python-Based Algorithm for Fitting the Bi-Exponential Model to Smoothed and Interpolated Actual Data. *Note*: The presented data are based on the results from a participant serving as a representative example for illustration purposes (see Appendix 1, time22 and oxy22 data for participant 22).
