# Supplementary material for: Enhancing Post‐Exercise Oxygen Kinetics Modeling With Physiological Bounds and Manual V̇O2_baseline Input: A Novel Approach
Source: Eur J Sport Sci. 2025 Apr 22;25(5):e12306. doi: 10.1002/ejsc.12306 (PMC12013733; doi:10.1002/ejsc.12306)
Supplement: Supplementary file 5 — Supporting Information S5 [file EJSC-25-e12306-s003.pdf]

```
import os
import pandas as pd
import numpy as np
from scipy.optimize import curve_fit
from scipy.interpolate import CubicSpline
import matplotlib.pyplot as plt
from scipy.integrate import trapz
from numpy.linalg import det


def get_file_path():
    directory_path = input("Please enter the directory path: ").replace('\\', '/')
    file_name = input("Please enter the file name: ")
    return os.path.join(directory_path, file_name)


def validate_file_path(full_file_path):
    return os.path.exists(full_file_path) and full_file_path.endswith('.xlsx')


def load_excel_data(full_file_path):
    return pd.read_excel(full_file_path)


def get_column_names():
    time_column = input("Enter the name of the time column: ")
    VO2_column = input("Enter the name of the oxygen column: ")
    return time_column, VO2_column


def mono_exp(t, VO2baseline, A1, td, tau1):
    return VO2baseline + A1 * np.exp(-(t - td) / tau1)


def bi_exp(t, VO2baseline, A1, td, tau1, A2, tau2):
    return VO2baseline + A1 * np.exp(-(t - td) / tau1) + A2 * np.exp(-(t - td) / tau2)
```

```
def slow_phase(t, VO2baseline, A2, td, tau2):
    return VO2baseline + A2 * np.exp(-(t - td) / tau2)


def five_point_smoothing(data):
    smoothed = data.copy()
    for i in data.index[2:-2]:
        try:
            smoothed.at[i] = (data.at[i-2] + data.at[i-1] + data.at[i] + data.at[i+1] + data.at[i+2]) / 5
        except KeyError:
            smoothed.at[i] = data.at[i]
    return smoothed


# Calculate the integral area
def calculate_integral(time, data):
    time_minute = time / 60
    data_litre = data / 1000
    return trapz(data_litre, time_minute)


# Get and validate file path and name
file_path = get_file_path()
if not validate_file_path(file_path):
    print("Invalid file path or file type")
    exit()


# Load Excel data
df = load_excel_data(file_path)


# Get column names
time_column, VO2_column = get_column_names()


# Extract data from the pandas DataFrame
```

```
time = df[time_column]

# Convert time to seconds
def time_to_seconds(time_str):
    if pd.isna(time_str) or time_str in ['nan', '']:
        return np.nan
    time_str = time_str.replace("saat: ", "")
    time_parts = list(map(int, time_str.split(':')))
    if len(time_parts) == 3:
        h, m, s = time_parts
        return h * 3600 + m * 60 + s
    elif len(time_parts) == 2:
        m, s = time_parts
        return m * 60 + s
    else:
        raise ValueError("Geçersiz zaman formatı.")

# Adjust oxygen values (replace comma with period)
df[VO2_column] = df[VO2_column].apply(lambda x: float(str(x).replace(",", ".")))

# Convert the time column to seconds
df[time_column] = df[time_column].apply(lambda x: time_to_seconds(str(x)))

# Filter out duplicate time values
df = df.loc[df.groupby(time_column)[VO2_column].idxmax()]

# Subtract the first value from the time data to set the start to zero
start_time = df[time_column].iloc[0]
df[time_column] = df[time_column] - start_time

time = df[time_column]
```

```
oxygen = df[VO2_column]

# Ask if user wants to apply smoothing to Oxygen data
smooth = input("Do you want to apply smoothing to oxygen data? (Y/N):: ")
if smooth.lower() == "y":
    oxygen = five_point_smoothing(oxygen)

# Ask if user wants to interpolate the data
interpolate = input("Do you want to apply interpolation to the data? (Y/N): ")
if interpolate.lower() == "y":
    cs = CubicSpline(time, oxygen)
    time = np.linspace(time.min(), time.max(), 500)
    oxygen = cs(time)

# Get VO2baseline value from user
VO2baseline_input = float(input("Please enter the VO2baseline value: "))

# Initialize bounds
mono_bounds = ([0, 0, 0], [np.inf, np.inf, np.inf])
bi_bounds = ([0, 0, 0, 0, 0], [np.inf, np.inf, np.inf, np.inf, np.inf])

# Use different bounds values if the user inputs a value between 100-1000 for absolute (mL/min) or between 1-10 for relative (mL/min/Kg)
if 100 <= VO2baseline_input <= 1000:
    mono_bounds = ([1000, 0, 20], [10000, 20, 180])
    bi_bounds = ([1000, 0, 15, 200, 120], [10000, 15, 90, 2000, 900])
elif 1 <= VO2baseline_input <= 10:
    mono_bounds = ([10, 0, 20], [100, 15, 180])
    bi_bounds = ([10, 0, 15, 2, 120], [100, 20, 90, 50, 900])
else:
    print("Invalid VO2baseline value. Please enter a value between 1-1000.")
```

```
    exit()

# Fit the models, using bounds
params1, _ = curve_fit(lambda t, A1, td, tau1: mono_exp(t, VO2baseline_input, A1, td, tau1), time, oxygen, bounds=mono_bounds)
params2, _ = curve_fit(lambda t, A1, td, tau1, A2, tau2: bi_exp(t, VO2baseline_input, A1, td, tau1, A2, tau2), time, oxygen, bounds=bi_bounds)

# Filter data for the first 5 minutes (300 seconds)
time_5min = time[time <= 300]
oxygen_5min = oxygen[time <= 300]

# Calculate R-squared for the whole model
y_pred1 = mono_exp(time, VO2baseline_input, *params1)
y_pred2 = bi_exp(time, VO2baseline_input, *params2)

rsq1 = 1 - (np.sum((oxygen - y_pred1)**2) / ((len(oxygen) - 1) * np.var(oxygen, ddof=1)))
rsq2 = 1 - (np.sum((oxygen - y_pred2)**2) / ((len(oxygen) - 1) * np.var(oxygen, ddof=1)))

# Calculate R-squared for the first 5 minutes
y_pred1_5min = mono_exp(time_5min, VO2baseline_input, *params1)
y_pred2_5min = bi_exp(time_5min, VO2baseline_input, *params2)

rsq1_5min = 1 - (np.sum((oxygen_5min - y_pred1_5min)**2) / ((len(oxygen_5min) - 1) * np.var(oxygen_5min, ddof=1)))
rsq2_5min = 1 - (np.sum((oxygen_5min - y_pred2_5min)**2) / ((len(oxygen_5min) - 1) * np.var(oxygen_5min, ddof=1)))

# AIC and AICc Calculation Function
def calculate_aic_aicc(n, mse, num_params):
    aic = n * np.log(mse) + 2 * num_params
    aicc = aic + (2 * num_params * (num_params + 1)) / (n - num_params - 1)
```

```
    return aic, aicc

# Fit the models, using bounds
params1, pcov1 = curve_fit(lambda t, A1, td, tau1: mono_exp(t, VO2baseline_input, A1, td, tau1), time, oxygen, bounds=mono_bounds)
params2, pcov2 = curve_fit(lambda t, A1, td, tau1, A2, tau2: bi_exp(t, VO2baseline_input, A1, td, tau1, A2, tau2), time, oxygen, bounds=bi_bounds)

# Calculate MSE for each model
mse1 = np.mean((oxygen - mono_exp(time, VO2baseline_input, *params1))**2)
mse2 = np.mean((oxygen - bi_exp(time, VO2baseline_input, *params2))**2)

# Calculate AIC and AICc
n = len(time)  # Number of data points

aic1, aicc1 = calculate_aic_aicc(n, mse1, len(params1))
aic2, aicc2 = calculate_aic_aicc(n, mse2, len(params2))

# Unit conversions
def ml_to_liter(ml):
    return ml / 1000

def seconds_to_minutes(seconds):
    return seconds / 60

def liter_to_kcal(litre):
    return litre * 5

def kcal_to_kjoule(kcal):
    return kcal * 4.184

# Calculate the integral of the models and the real data
```

```
integral_real_data = calculate_integral(time, oxygen)
integral_mono_model = calculate_integral(time, y_pred1)
integral_bi_model = calculate_integral(time, y_pred2)

# Calculate energy production for the integrals
integral_energy_real_kcal = liter_to_kcal(integral_real_data)
integral_energy_real_kjoule = kcal_to_kjoule(integral_energy_real_kcal)

integral_energy_mono_kcal = liter_to_kcal(integral_mono_model)
integral_energy_mono_kjoule = kcal_to_kjoule(integral_energy_mono_kcal)

integral_energy_bi_kcal = liter_to_kcal(integral_bi_model)
integral_energy_bi_kjoule = kcal_to_kjoule(integral_energy_bi_kcal)

# Add a function to filter data for the first 5 minutes
def filter_first_five_minutes(time, data):
    time_5_min_index = time <= 300  # 300 seconds equals 5 minutes
    return time[time_5_min_index], data[time_5_min_index]

# Filter the data for the first 5 minutes
time_5_min, oxygen_5_min = filter_first_five_minutes(time, oxygen)
_, y_pred1_5_min = filter_first_five_minutes(time, y_pred1)
_, y_pred2_5_min = filter_first_five_minutes(time, y_pred2)

# Calculate the integral for the first 5 minutes
integral_real_data_5_min = calculate_integral(time_5_min, oxygen_5_min)
integral_mono_model_5_min = calculate_integral(time_5_min, y_pred1_5_min)
integral_bi_model_5_min = calculate_integral(time_5_min, y_pred2_5_min)

# Calculate energy production for the first 5 minutes
integral_energy_real_5_min_kcal = liter_to_kcal(integral_real_data_5_min)
```

```
integral_energy_real_5_min_kjoule = kcal_to_kjoule(integral_energy_real_5_min_kcal)

integral_energy_mono_5_min_kcal = liter_to_kcal(integral_mono_model_5_min)
integral_energy_mono_5_min_kjoule = kcal_to_kjoule(integral_energy_mono_5_min_kcal)

integral_energy_bi_5_min_kcal = liter_to_kcal(integral_bi_model_5_min)
integral_energy_bi_5_min_kjoule = kcal_to_kjoule(integral_energy_bi_5_min_kcal)

# Convert A1 and tau1 values
A1_mono_liter = ml_to_liter(params1[0])
tau1_mono_minute = seconds_to_minutes(params1[2])

A1_bi_liter = ml_to_liter(params2[0])
tau1_bi_minute = seconds_to_minutes(params2[2])

# Calculate oxygen consumption
O2_mono = A1_mono_liter * tau1_mono_minute
O2_bi = A1_bi_liter * tau1_bi_minute

# Calculate energy production
energy_mono_kcal = liter_to_kcal(O2_mono)
energy_mono_kjoule = kcal_to_kjoule(energy_mono_kcal)

energy_bi_kcal = liter_to_kcal(O2_bi)
energy_bi_kjoule = kcal_to_kjoule(energy_bi_kcal)

# Print the parameter outputs
print(f"\nMono-exponential model parameters:")
print(f"R-squared: {rsq1:.3f}")
print(f"VO2baseline = {VO2baseline_input:.3f}, A1 = {params1[0]:.3f}, td = {params1[1]:.3f}, tau1 = {params1[2]:.3f}")
```

```
print(f"\nBi-exponential model parameters:")

print(f"R-squared: {rsq2:.3f}")

print(f"VO2baseline = {VO2baseline_input:.3f}, A1 = {params2[0]:.3f}, td = {params2[1]:.3f}, tau1 =
{params2[2]:.3f}, A2 = {params2[3]:.3f}, tau2 = {params2[4]:.3f}")


# Print the Akaike information criterion-AIC

print(f"\nMono-exponential model AIC: {aic1:.3f}, AICc: {aicc1:.3f}")

print(f"Bi-exponential model AIC: {aic2:.3f}, AICc: {aicc2:.3f}")


# Print the energy outputs

print(f"\nMono-exponential model for energy contribution of alactic pathway:")

print(f"{O2_mono:.2f} liter O2 = {energy_mono_kcal:.2f} kcal = {energy_mono_kjoule:.2f} kJ")


print(f"\nBi-exponential model for energy contribution of alactic pathway:")

print(f"{O2_bi:.2f} liter O2 = {energy_bi_kcal:.2f} kcal = {energy_bi_kjoule:.2f} kJ")


# Print the integral outputs

print(f"\nIntegral of real data:")

print(f"{integral_real_data:.2f} liter O2 = {integral_energy_real_kcal:.2f} kcal =
{integral_energy_real_kjoule:.2f} kJ")


print(f"\nIntegral of Mono-exponential model:")

print(f"{integral_mono_model:.2f} liter O2 = {integral_energy_mono_kcal:.2f} kcal =
{integral_energy_mono_kjoule:.2f} kJ")


print(f"\nIntegral of Bi-exponential model:")

print(f"{integral_bi_model:.2f} liter O2 = {integral_energy_bi_kcal:.2f} kcal =
{integral_energy_bi_kjoule:.2f} kJ")


# Print the integral outputs for the first 5 minutes

print(f"\nIntegral of real data for the first 5 minutes:")
```

```
print(f"{integral_real_data_5_min:.2f} liter O2 = {integral_energy_real_5_min_kcal:.2f} kcal = {integral_energy_real_5_min_kjoule:.2f} kJ")


print(f"\nIntegral of Mono-exponential model for the first 5 minutes:")

print(f"{integral_mono_model_5_min:.2f} liter O2 = {integral_energy_mono_5_min_kcal:.2f} kcal = {integral_energy_mono_5_min_kjoule:.2f} kJ")


print(f"\nIntegral of Bi-exponential model for the first 5 minutes:")

print(f"{integral_bi_model_5_min:.2f} liter O2 = {integral_energy_bi_5_min_kcal:.2f} kcal = {integral_energy_bi_5_min_kjoule:.2f} kJ")


# Print R-squared for the first 5 minutes

print(f"\nMono-exponential model R-squared for first 5 minutes: {rsq1_5min:.3f}")

print(f"Bi-exponential model R-squared for first 5 minutes: {rsq2_5min:.3f}")


# Plot the graphs

plt.figure(figsize=(10, 4))


# Graph for Mono-exponential model

plt.figure(figsize=(10, 4))

plt.scatter(time, oxygen, label='Actual data', s=5)

plt.plot(time, y_pred1, label='Mono-exponential fit', color='r', linewidth=2)

plt.axhline(y=VO2baseline_input, color='purple', linestyle='--', label='VO2baseline')

plt.title('Mono-exponential Model', fontname="Times New Roman")

plt.xlabel('Time (s)', fontname="Times New Roman")

plt.xticks(np.arange(0, max(time)+1, 60), fontname="Times New Roman")

if max(oxygen) <= 500:

   step = 5

   label = 'Oxygen (mL/min/Kg)'

else:

   step = 500

   label = 'Oxygen (mL/min)'
```

```
plt.yticks(np.arange(0, max(oxygen)+1, step), fontname="Times New Roman")

plt.ylabel(label, fontname="Times New Roman")

plt.legend(fontsize='small', loc='best')

plt.grid(True)

plt.gca().set_facecolor('white')

plt.tight_layout()


# Save the Mono-exponential model graph as JPEG with 300 DPI

plt.savefig('mono_exponential_graph.jpg', dpi=300, format='jpeg')


# Graph for fast and slow phases of Bi-exponential model

plt.figure(figsize=(10, 4))

plt.scatter(time, oxygen, label='Actual data', s=5)

plt.plot(time, y_pred2, label='Fast phase', color='r', linewidth=2)

plt.plot(time, slow_phase(time, VO2baseline_input, params2[3], params2[1], params2[4]), 'g-.',
label='Slow phase', linewidth=2)

plt.axhline(y=VO2baseline_input, color='purple', linestyle='--', label='VO2baseline')

plt.title('Bi-exponential Model', fontname="Times New Roman")

plt.xlabel('Time (s)', fontname="Times New Roman")

plt.xticks(np.arange(0, max(time) + 1, 60), fontname="Times New Roman")

if max(oxygen) <= 500:

   step = 5

   label = 'Oxygen (mL/min/Kg)'

else:

   step = 500

   label = 'Oxygen (mL/min)'


plt.yticks(np.arange(0, max(oxygen) + 1, step), fontname="Times New Roman")

plt.ylabel(label, fontname="Times New Roman")

plt.grid(True)
```

```
plt.legend(fontsize='small', loc='best')

plt.gca().set_facecolor('white')

plt.tight_layout()


# Save the Bi-exponential model graph as JPEG with 300 DPI

plt.savefig('bi_exponential_graph.jpg', dpi=300, format='jpeg')
```
